# EPBAM: annotation-robust detection of alternative splicing events and a systematic benchmarking of current tools

**DOI:** 10.1093/bib/bbag556

**Published:** 2026-10-08

**Authors:** Cesar Lobato-Fernandez, Laura Jareno, Angel Rubio, Juan Angel Ferrer-Bonsoms

**Affiliations:** Biomedical Engineering and Science Department, TECNUN, University of Navarra, Paseo de Manuel Lardizábal 13, 20018 Donostia-San Sebastián, Gipuzkoa, Spain; Biomedical Engineering and Science Department, TECNUN, University of Navarra, Paseo de Manuel Lardizábal 13, 20018 Donostia-San Sebastián, Gipuzkoa, Spain; Biomedical Engineering and Science Department, TECNUN, University of Navarra, Paseo de Manuel Lardizábal 13, 20018 Donostia-San Sebastián, Gipuzkoa, Spain; Biomedical Engineering and Science Department, TECNUN, University of Navarra, Paseo de Manuel Lardizábal 13, 20018 Donostia-San Sebastián, Gipuzkoa, Spain

**Keywords:** alternative splicing, SIRV, bootstrap based statistics, bioinformatics, RNA splicing

## Abstract

Alternative splicing (AS) is a major driver of transcriptomic diversity, yet its computational analysis remains challenged by inconsistent event definitions across tools and by the sensitivity of quantification methods to annotation quality. Here, we present a comprehensive benchmark of seven AS detection tools—replicate Multivariate Analysis of Transcript Splicing (rMATS), Super-fast Pipeline for alternative splicing analysis, version 2 (SUPPA2), Modeling Alternative Junction Inclusion Quantification + Visualization Of Inferred Local splicing Alternatives (MAJIQ+VOILA), EventPointerST (EPST), Shiba, LeafCutter, and the newly introduced EventPointer BAM (EPBAM)—evaluated across three complementary datasets: reverse transcription PCR (RT-PCR) validated experiments, simulated data, and Lexogen Spike-In RNA Variant (SIRV). The inclusion of SIRV, which provide experimentally derived sequencing data with fully controlled ground truth, is central to our evaluation framework, enabling rigorous assessment of quantification accuracy under real technical conditions. We assess tool performance under three annotation scenarios—complete, incomplete, and overloaded—reflecting the annotation uncertainty commonly encountered in practice. EPBAM extends the EventPointer framework by enabling *de novo* event detection coupled with a coverage-corrected Ψ quantification strategy. Our results demonstrate that annotation quality substantially drives tool performance: annotation-dependent tools achieve peak accuracy under complete annotations, whereas EPBAM exhibits the most robust and consistent behaviour across annotation conditions, recovering unannotated events with low false discovery rates. No single tool dominates across all evaluation metrics, highlighting the importance of dataset diversity and standardized benchmarking practices for meaningful tool comparison in the AS field.

## Graphical Abstract

**Graphical Abstract:**
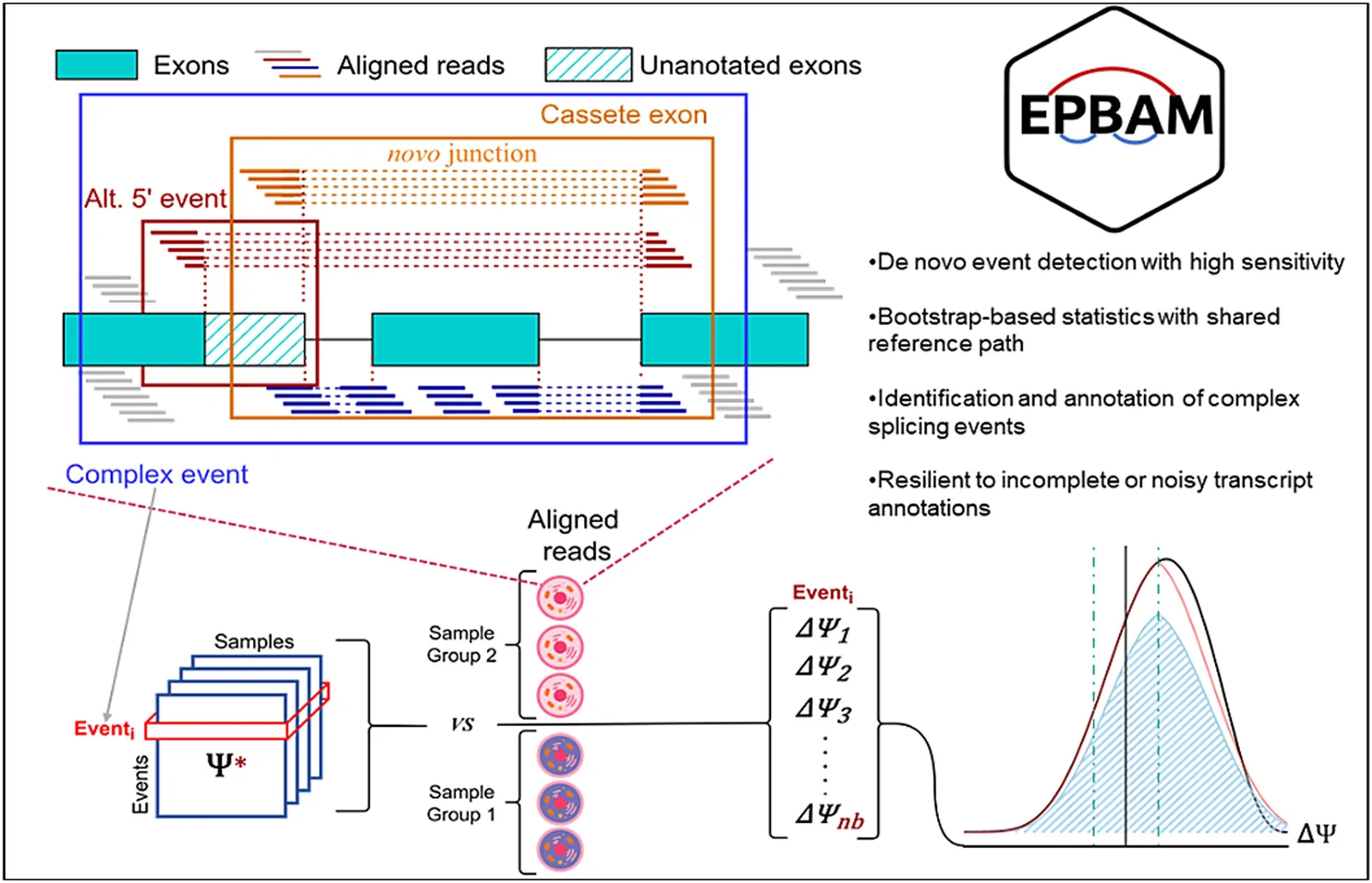

## Introduction

Alternative splicing (AS) is a fundamental mechanism in eukaryotic gene expression that enables the production of multiple mature messenger RNA (mRNA) variants from a single gene through alternative splice site selection. This process increases transcriptomic complexity and functionality and plays a crucial role in cellular differentiation, development, and adaptability [1]. The regulatory networks governing AS are finely tuned to respond to developmental cues, environmental changes, and cellular states, and aberrant splicing patterns have been linked to a wide range of human diseases, including cancer, muscular dystrophies, and neurodegenerative disorders [1–4].

High-throughput RNA sequencing (RNA-seq) provides sensitive and versatile measurements of gene expression [5]. However, accurately quantifying full-length transcript isoforms from short-read RNA-seq is challenging [6, 7], particularly in the presence of unannotated or novel isoforms [8–10]. As a result, many computational approaches focus on local AS events rather than on potentially error-prone inference of complete isoform structures and abundances [11].

A splicing event is commonly defined as a localized alteration in pre-mRNA processing, such as the use of alternative splice donor or acceptor sites or the inclusion or exclusion of specific regions within the transcript [12]. These events are typically quantified using the percent spliced in (Ψ) metric, which estimates the relative abundance of isoforms that include an AS path compared with the total expression at a given locus. While this event-centric framework provides a tractable way to study AS, the definition of what constitutes a ‘splicing event’ is broad and inherently subjective. Different tools adopt distinct conceptualizations and labelling schemes—ranging from a restricted set of canonical patterns to complex multi-exon rearrangements—hindering consistent benchmarking and direct comparison across methods.

Canonical AS events are the standard patterns commonly described in eukaryotic transcriptomes. These include cassette exons (CEs) (exon skipping), intron retention, alternative 5′ splice sites, alternative 3′ splice sites, and mutually exclusive exons (MXE). In addition, although they arise from changes at the level of transcription rather than splicing per se, alternative last exons and alternative first exons are often considered within the same conceptual framework [3, 13]. Detailed descriptions and graphical representations of these canonical events are provided in Supplementary Fig. S1 and Supplementary Text S1 (Supplementary File 1). However, gene splicing patterns are often too complex to be fully captured by canonical categories alone. Many software tools therefore introduce ‘complex events’ [14] as a catch-all class for local patterns that do not fit any canonical type.

These conceptual ambiguities in event definition are compounded by technical challenges in Ψ and differential splicing (ΔΨ) estimation. Bootstrap-based strategies, especially those leveraging uncertainty estimates from pseudo-aligners such as Kallisto or Salmon, have been shown to perform well for Ψ estimation and for assessing ΔΨ uncertainties [15]. However, these approaches are limited to annotated events, and their extension to *de novo* events is not straightforward, since most strategies based on splice-aware mapping do not provide bootstrap information.

In addition, Ψ and ΔΨ estimates are sensitive to read coverage biases, mapping uncertainties, and modelling assumptions [16]. Wet-lab validation strategies, while useful to identify true and false positives among detected events, provide limited information on false negatives. Further, these validations do not allow a systematic assessment of which factors—such as the event definition or the underlying statistical model—most strongly influence performance. Therefore, there is a clear need for independent, systematic evaluations that explicitly consider formal event definitions and statistical modelling choices.

Here, we introduce EPBAM, a new tool for splicing analysis integrated into the EventPointer R package. EPBAM relies on splice-aware aligners to (i) detect novel splicing events and (ii) construct a bootstrap-based framework for estimating Ψ and ΔΨ. This bootstrap model operates at the read level, effectively augmenting splice-aware aligners with bootstrap-derived uncertainty estimates analogous to those provided by pseudo-aligners such as Kallisto and Salmon. In this way, EPBAM is able to detect *de novo* canonical and complex splicing events while maintaining statistical robustness.

We evaluated EPBAM alongside the main AS detection algorithms replicate Multivariate Analysis of Transcript Splicing (rMATS) [17–19], Super-fast Pipeline for alternative splicing analysis, version 2 (SUPPA2) [20–24], Modeling Alternative Junction Inclusion Quantification + Visualization Of Inferred Local splicing Alternatives (MAJIQ+VOILA) [21–26], Shiba, LeafCutter, and EventPointer ST (EPST) [15] using Lexogen Spike-In RNA Variant (SIRV) controls [27], which provide synthetic transcripts with known composition and defined Ψ and ΔΨ values, thereby offering an externally validated ground truth for benchmarking. This allowed us to assess detection performance, quantification accuracy, and the impact of annotation completeness under controlled yet realistic conditions. In addition, we complemented this analysis with simulated RNA-seq data and real biological datasets, enabling a comprehensive evaluation of method behaviour across a range of experimental and biological scenarios.

Beyond introducing EPBAM, this work provides a focused review of current AS event detection tools, clarifying how differences in event definitions and modelling strategies translate into divergent analytical outcomes. Our results reveal substantial discrepancies between tools, driven not only by differences in quantification or statistical frameworks but also by fundamentally distinct assumptions about the nature and representation of splicing events. In this context, EPBAM demonstrates competitive performance with particular robustness to annotation quality, and our findings underscore the need for more standardized practices in AS event representation and benchmarking.

### Overview of alternative splicing software

Event-based AS tools differ along several key dimensions that critically affect their behaviour and output. First, they rely on distinct RNA-seq processing strategies, using either splice-aware aligners or transcript-level pseudo-aligners as their primary input. Second, they adopt different formal definitions of what constitutes an AS event and implement diverse algorithms to detect these events from the underlying read or junction information. Third, they use alternative schemes to quantify events, typically via Ψ or related quantities, which may be derived from junction counts or transcript abundances. Finally, they apply different statistical frameworks to test for differential splicing, ranging from simple count-based models to more elaborate bootstrap or Bayesian approaches.

Alternative approaches to event-based AS analysis also exist, such as the feature-based methods implemented in tappAS [28] or SGSeq [29], or more minimalist strategies like LeafCutter [30], which identifies differential splicing through intron excision clusters rather than predefined events. More recently, Shiba [31] has been proposed as another tool for splicing analysis. However, LeafCutter’s intron-cluster-based event representation does not map directly onto the path-pair framework used by EventPointer, rMATS, or SUPPA2, making quantitative comparison of Ψ values across tools problematic. An exhaustive evaluation of all available methods lies beyond the scope of this work; we focus on tools whose event definitions enable systematic cross-tool comparison of Ψ and ΔΨ estimates.

#### Sequence alignment

RNA-seq alignment is a critical step in AS analysis. Some tools—such as SUPPA2 and EPST—utilize pseudo-aligners, which have gained traction for their computational efficiency and versatility [32, 33]. In contrast, tools leveraging splice-aware aligners [34–37], such as MAJIQ+VOILA, rMATS, EPBAM, LeafCutter, Shiba, SGSeq, ASpli [38], and Alternative Splicing Graph ALigner (ASGAL) [39], facilitate *de novo* event detection, enabling the identification of novel splicing patterns in various biological contexts. As pseudo-aligners only estimate transcript or isoform abundances, methods that rely on them are affected by alignment choices primarily at the level of event quantification. By contrast, for splice-aware-based methods the alignment step influences both event detection and quantification.

#### Alternative splicing event definition and detection

Understanding how tools define and detect AS events first requires introducing the concept of a splicing graph (SG). An SG is a directed acyclic graph that models the AS patterns of a gene. In its most common form, nodes represent the genomic positions of splice sites, whereas edges correspond to exons or junction reads that connect these sites [40]. Constructing an SG, either explicitly or implicitly, is therefore a critical step in splicing analysis, as it underlies the detection and classification of splicing events. Methods that use splice-aware aligners build their corresponding SG from the output of these aligners; consequently, the SG can capture sample-specific structures, enabling the detection of *de novo* events. In contrast, methods that operate on a reference transcriptome derive SGs from annotated isoforms, and therefore their graphs primarily represent known, annotated structures.

Each tool leverages this concept differently. rMATS, SUPPA2, and Shiba primarily rely on exon inclusion/exclusion patterns to define canonical events. MAJIQ+VOILA conceptualizes splicing in terms of Local Splicing Variations (LSVs), which represent branch points in a splice graph where multiple paths originate or converge, and are used to categorize both canonical and complex patterns. EPST and EPBAM construct a complete SG for each gene and define events through three paths—an alternative path, a constitutive path, and a shared reference path—enabling the identification of complex events beyond canonical patterns (Supplementary Fig. S2). On the other hand, LeafCutter also builds an SG, but with a different convention: introns are the nodes and edges connect introns that share a splice site. The connected components of this intron-based SG define intron clusters, which, after iteratively removing rarely used introns, constitute LeafCutter’s unit of analysis in place of a discretely labelled AS event. Because a cluster can contain an arbitrary number of alternatively excised introns, this representation captures complex, multi-way splicing choices without requiring them to be classified into canonical categories such as skipping exon (SE), alternative 5’ splicing site, alternative 3’ splicing site, retained intron (RI), or MXE, and—since clusters are built exclusively from observed split reads—it applies identically to annotated and novel excision patterns (Supplementary Fig. S3). More details on event definitions are provided in Supplementary Text S1.

#### Quantification of alternative splicing events

In most methods, the quantification of events is performed using Ψ. The Ψ can be calculated with the following formula:


(1) \begin{eqnarray*} \Psi =\frac{\boldsymbol{A}}{\boldsymbol{A}+\boldsymbol{B}} \end{eqnarray*}

where A and B represent the abundances of the alternative and constitutive splice forms, respectively. This metric quantifies the relative prevalence of a specific splicing choice at a given locus. Thus, the main differences between methods lie in how A and B are estimated.

Methods that rely on pseudo-aligners exploit transcript-level expression estimates. In these tools, A and B are obtained by summing the expression of the transcripts that contain the alternative and constitutive splice forms, respectively. For this reason, they are often referred to as isoform-based tools. In contrast, alignment-based methods such as rMATS, EPBAM, and Shiba compute Ψ directly from reads mapped to the genomic region of the splicing event. In rMATS and EPBAM, A is estimated as I/L_i_ and B as S/L_j_, where I and S denote the counts of reads that include or exclude the event, and L_i_ and L_j_ represent the effective lengths of the corresponding regions. Shiba follows the same underlying logic but restricts A and B to exon-exon and exon-intron junction-spanning read counts: because every junction read spans a splice site regardless of the length of the flanking exon or intron, all junction reads share the same effective length, so no explicit length correction is required.

Two methods depart substantially from this two-path formulation: LeafCutter and MAJIQ+VOILA. LeafCutter does not define a fixed alternative and constitutive path at all; instead, for each intron within a cluster of alternatively excised introns, it reports the proportion of the cluster’s total junction reads that support that intron. When a cluster contains exactly two introns, this proportion reduces to the same A/(A + B) form used above, but for clusters with three or more introns it yields one Ψ value per intron rather than a single Ψ for the cluster.

MAJIQ+VOILA, in turn, quantifies an event by modelling LSVs as junction-level events (Supplementary Fig. S9). In canonical cases, this quantification is derived from the relative inclusion level of a junction; for complex events, it averages the marginal inclusion levels of all junctions within a given path. The full summarization approach is detailed in Supplementary Text S3.

#### Statistical analysis of splicing

EPBAM, EPST, SUPPA, and rMATS effectively estimate the distribution of the ΔΨ to determine whether it differs significantly from zero, whereas MAJIQ+VOILA, Shiba, and LeafCutter do not.

SUPPA2 infers significance by comparing observed ΔΨ values to a background empirical distribution constructed from transcripts of similar abundance. False discovery rate (FDR) correction is applied to adjust for multiple testing. Although fast and annotation-based, SUPPA2 does not include ΔΨ thresholds in its core testing model, but these can be applied post hoc.

rMATS models Ψ values with a binomial distribution and uses a hierarchical framework to capture replicate variability. Significance is tested via a likelihood ratio test (LRT), incorporating a user-defined ΔΨ threshold to prioritize biologically meaningful changes.

EPST uses a bootstrap-based strategy to estimate the distribution of ΔΨ. For each bootstrap sample returned by the pseudo-aligner, EPST first computes a Ψ value; these Ψ estimates are then combined using bootstrap resampling across samples to obtain an empirical distribution of ΔΨ. EPBAM adapts this idea of empirical estimation of the ΔΨ distribution but performs bootstrap resampling at the read level over alignments mapping to the genomic region of the event. A more detailed description of this procedure is provided in the Methods section.

MAJIQ+VOILA adopts a Bayesian framework to estimate the posterior distribution of Ψ for each junction, from which ΔΨ values and associated confidence measures are derived. Although MAJIQ+VOILA and EPST both exploit resampling-based uncertainty, their interpretations differ: MAJIQ quantifies the probability of a significant change based on user-defined thresholds, whereas EPST computes *P*-values from the confidence interval around ΔΨ (Supplementary Fig. S10).

Shiba departs from these distribution-based approaches; it tests each event directly on pooled junction read counts using Fisher’s exact test on the corresponding inclusion/exclusion contingency tables. Shiba+ extends this test by additionally requiring a significant Welch’s *t-*test on the per-sample Ψ values, incorporating replicate variability that the pooled contingency-table test alone does not capture.

LeafCutter tests each intron cluster as a whole: intron counts within a cluster are jointly modelled with a Dirichlet-multinomial generalized linear model, and a LRT evaluates whether the group covariate explains variation in intron usage across the cluster. This yields a single *P*-value per cluster rather than per individual intron or canonical event type (see Supplementary Text S4 for further details).

Table 1 summarizes the main methods for AS analysis. In this work, we focus on tools that study local splicing changes, often employing the concept of AS events, as discussed above.

**Table 1:** Comparative analysis of AS software tools for RNA-seq data analysis.

Software		rMATS	SUPPA2	MAJIQ + VOILA	Shiba	LeafCutter	SplAdder	SGSeq	Whippet	ASpli	ASGAL	EPST	EPBAM	tappAS	AS-Quant	McSplicer	VAST-TOOLS
OS		Linux	Linux	Linux	Linux	All	All	All	All	All	Linux	All	All	All	Linux	All	Linux
**Events**		✔️	✔️	✔️	✔️	×	✔️	✔^*^	✔️	✔️	✔️	✔️	✔️	✔^*^	✔️	✔️	✔️
Ψ		✔️	✔️	✔^*^	✔️	✔^*^	✔️	✔️	✔️	✔️	×	✔️	✔️	✔^*^	×	✔️	✔️
**Differential AS statistics**		✔️	✔️	✔^*^	✔️^*^	✔^*^	✔️	×	✔️	×	×	✔️	✔️	✔^*^	×	×	✔️
**Last update**		2024	2024	2024	2026	2023	2024	2020	2023	2021	2024	2025	2026	2023	2023	2022	2024
**Event detection**	SG for a specific event/region	✔️	✔️	×	×	×	×	✔️	×	✔️	×	×	×	×	✔️	×	×
	Complete gene SG	×	×	✔️	✔️	×	✔️	✔️	✔️	×	✔️	✔️	✔️	✔️	×	✔️	✔️
	GTF-based event detection	✔️	✔️	×	✔️	×	✔️	✔️	✔️	✔️	✔️	✔️	×	✔️	✔️	✔️	✔️
	*De novo* event detection	✔️	×	✔️	✔️	✔️	×	✔️	×	✔️	✔️	×	✔️	×	×	×	×
	Splice-aware aligners	✔️	×	✔️	✔️	✔️	✔️	✔️	✔️	✔️	✔️	×	✔️	✔️	✔️	✔️	✔️
	Pseudo-aligners	×	✔️	×	×	×	×	×	×	×	✔️	✔️	×	×	×	×	×
**Labelling of the events**	Exon skipping (ES)	✔️	✔️	✔️	✔️	**–**	✔️	**–**	✔️	✔️	✔️	✔️	✔️	**–**	✔️	✔️	✔️
	Intronic retention (IR)	✔️	✔️	✔️	✔️	**–**	✔️	**–**	✔️	✔️	✔️	✔️	✔️	**–**	✔️	✔️	✔️
	Alternative 5’ splicing site	✔️	✔️	✔️	✔️	**–**	✔️	**–**	✔️	✔️	✔️	✔️	✔️	**–**	✔️	✔️	✔️
	Alternative 3’ splicing site	✔️	✔️	✔️	✔️	**–**	✔️	**–**	✔️	✔️	✔️	✔️	✔️	**–**	✔️	✔️	✔️
	Alternative first exon (AFE)	×	✔️	✔️	✔️	**–**	×	**–**	✔️	×	×	✔️	✔️	**–**	×	×	×
	Alternative last exon (ALE)	×	✔️	✔️	✔️	**–**	×	**–**	✔️	×	×	✔️	✔️	**–**	×	×	×
	MXE	✔️	✔️	✔️	✔️	**–**	✔️	**–**	✔️	×	×	✔️	✔️	**–**	✔️	✔️	✔️
	Complex events (e.g. multi-exon skipping)	×	×	✔️	✔️	**–**	×	**–**	✔️	×	×	✔️	✔️	**–**	×	✔️	✔️
Ref		[17–19]	[20]	[21–26]	[31]	[30]	[41]	[29]	[42]	[38]	[39]	[14, 15]	This paper	[28]	[37]	[43]	[44]

The table provides an overview of different features and capabilities of various AS tools. Key attributes compared are operating system compatibility, support for event-based AS detection, Ψ (percent spliced in) calculation, and differential AS statistics. Additional details include citation counts, last update year, and each tool’s ability to detect novel splicing events. Tools are further evaluated on their labelling of AS canonical and complex events. Tools that do not match exactly with the canonical definition of an event, Ψ or differential Ψ are marked with an asterisk (*).

Among the tools included in Table 1, we selected rMATS, EPST, SUPPA2, Shiba, LeafCutter, and MAJIQ for in-depth comparison with EPBAM. These seven tools collectively span the main methodological axes in AS software: alignment strategy (splice-aware for rMATS, MAJIQ+VOILA, Shiba, LeafCutter, and EPBAM versus pseudo-alignment for SUPPA2 and EPST), event complexity (canonical only for rMATS and SUPPA2 versus canonical and complex for EPST, EPBAM, and MAJIQ+VOILA), annotation dependency (annotation-constrained for SUPPA2 and EPST versus *de novo* capable for rMATS, MAJIQ+VOILA, Shiba, LeafCutter, and EPBAM), and statistical framework (frequentist versus Bayesian for MAJIQ+VOILA). rMATS, SUPPA2, and MAJIQ+VOILA are among the most widely cited tools in this field.

## Materials and methods

### EPBAM: event detection, quantification, and statistical framework

#### Event definition and detection

EPBAM constructs a SG that is conceptually equivalent to that used by EPST. In this graph, nodes represent the start and end of each subexon—contiguous genomic segment that is shared by the same subset of transcripts—and edges represent either contiguous exons/subexons or splice junctions (Fig. 1, panel 2). Thus, both EPST and EPBAM build an SG that fully represents the structure of each gene. Once the SG has been constructed, EPST and EPBAM use the same definition and procedure to detect events: each event is defined as being composed of three distinct paths, Path A, Path B, and a third path known as the reference path [14]. The reference path contains the regions shared by the isoforms that include either Path A or Path B within the SG (Supplementary Fig. S2D). This formulation allows the identification of complex events that extend beyond canonical splicing patterns. The main difference between EPST and EPBAM lies in the source of information used to build the SG: EPST relies on a reference transcriptome [15], whereas EPBAM derives the SG not only from the reference transcriptome but from the splice-aware alignments, enabling EPBAM to detect novel events (Fig. 1, panel 1 and 3). It is important to note that, while EPBAM constructs the splice graph from aligned reads without directly querying the annotation file, the upstream alignment step introduces an indirect dependency on the annotation. Specifically, Spliced Transcripts Alignment to a Reference (STAR) [34] uses the annotated splice junctions to build its genome index, which influences the mapping of reads spanning novel or ambiguous junctions. Reads supporting a novel junction that is not present in the annotation can still be mapped by STAR—this is what enables *de novo* event detection—but the efficiency of this mapping may vary depending on the annotation used. Consequently, EPBAM’s event detection is annotation-robust rather than annotation-independent: the annotation influences which reads are successfully aligned, even though EPBAM itself operates solely on the resulting alignments. This explains the small variations in the number of detected events observed across annotation conditions (see Results, Table 3).

**Figure 1:**
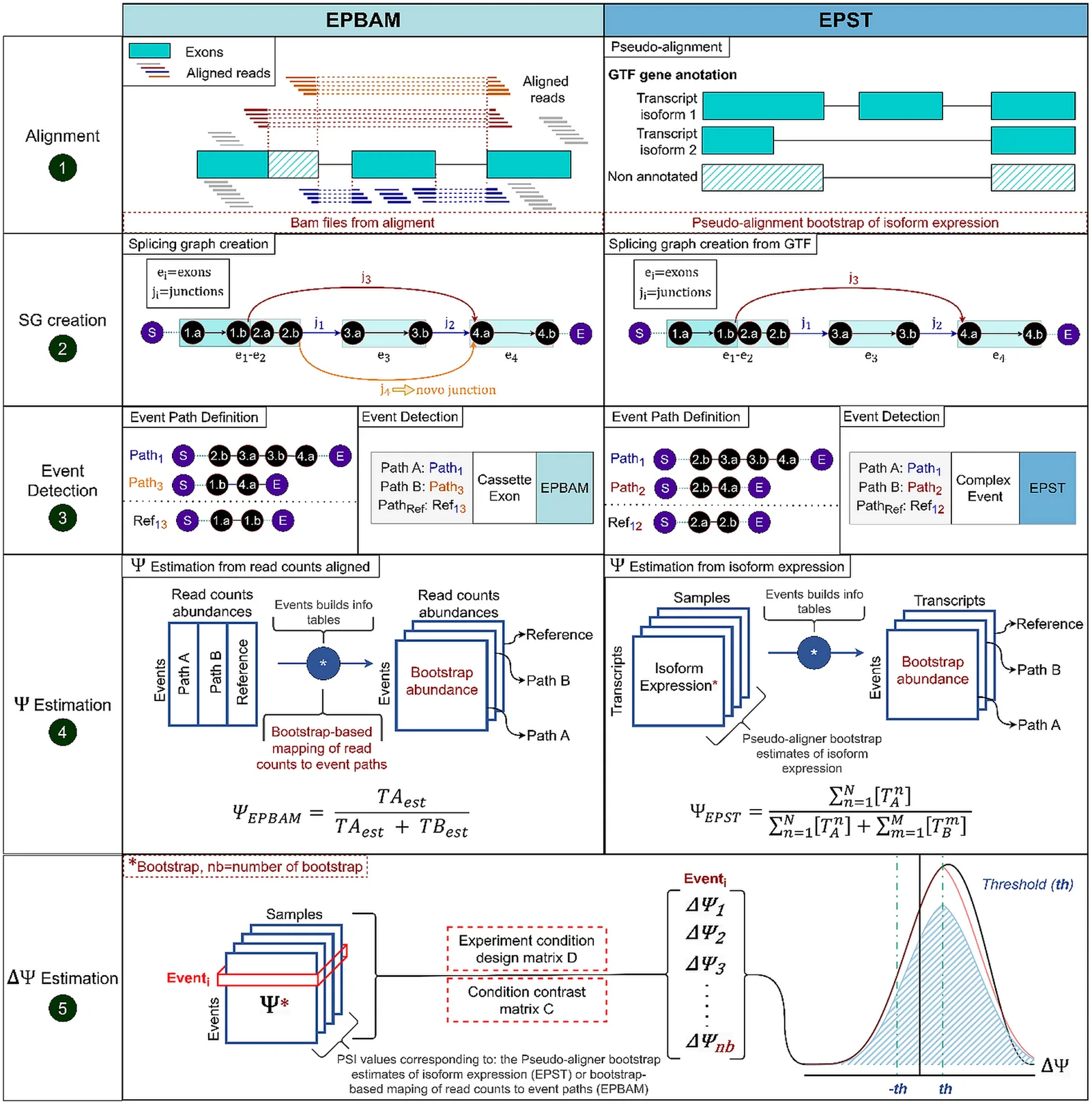
Workflow of EPBAM (first column) and EPST (second column) from the EventPointer software across the different phases of AS analysis; (1) alignment phase: EPBAM performs a splice-aware alignment, whereas EPST uses pseudo-alignment; (2) SG construction: both tools create an SG, differing only in the detection of *de novo* edge representing a junction between two exons; note that for EPBAM, the annotation indirectly influences the splice graph through the STAR alignment step (see methods); (3) event detection based on the constructed SG; (4) Ψ estimation: EPBAM estimates Ψ using read counts from the alignment associated with each path, while EPST derives it from transcript abundances; (5) ΔΨ estimation, common to both EPBAM and EPST.

#### Events quantification

As EPBAM is an alignment-based tool, PSI quantification follows a scheme like that of rMATS. Equation 1 for the calculation of Ψ can be rewritten as:


(2) \begin{eqnarray*}{\Psi}_{EPBAM}^{\ast }=\frac{R_A/{l}_A^{\ast }}{R_B/{l}_B^{\ast }+{R}_A/{l}_A^{\ast }}\end{eqnarray*}

where ${R}_A$ and ${R}_B$ are the read counts associated with each path, and ${l}_A^{\ast }$ and ${l}_B^{\ast }$ are the effective lengths associated with each path. Because EPBAM explicitly includes a reference path, which could be used directly to estimate the denominator of Equation 2:


(3) \begin{eqnarray*}{\Psi}_{EPBAM}^{\ast }=\frac{R_A/{l}_A^{\ast }}{R_{ref}/{l}_{ref}^{\ast }}\end{eqnarray*}

where ${R}_{ref}$ and ${l}_{ref}^{\ast }$ denote the read count and effective length of the reference path, respectively. However, sequencing efficiency is not uniform across loci: read coverage can vary due to guanine–cytosine (GC)-content biases, mappability differences, and local chromatin or sequence context effects. The classical approach—used by rMATS and other alignment-based tools—assumes that the concentration of isoforms in each path is simply proportional to its read count divided by a fixed effective length. This assumption can introduce systematic errors in Ψ estimation when coverage is uneven across the paths of an event. EPBAM addresses this by treating the effective lengths as unknown parameters to be estimated from the data, exploiting the redundancy provided by the reference path:


(4) \begin{eqnarray*}{R}_{ref}/{l}_{ref}^{\ast}\ne{R}_A/{l}_A^{\ast }+{R}_B/{l}_B^{\ast }\end{eqnarray*}

In an ideal scenario, the underlying concentration would satisfy:


(5) \begin{eqnarray*}\left[{TRef}_{est}\right]=\left[{TA}_{est}\right]+\left[{TB}_{est}\right],\end{eqnarray*}

where ${TRef_{est}}_i$, ${TA_{est}}_i$, and ${TB_{est}}_i$ are the real concentrations in the reference region, path A and B, respectively.

The key insight underlying EPBAM’s approach is that the reference path provides an independent estimate of the total isoform concentration at the locus. By definition, the reference path is shared by all isoforms that include either Path A or Path B (Equation 5). This means that the read count in the reference path, properly normalized, should equal the sum of the normalized read counts in Paths A and B. EPBAM exploits this redundancy by introducing a soft constraint that links the concentrations of the three paths, allowing the effective-length ratios to be estimated from the data rather than assumed a priori. To do so, EPBAM assumes that each concentration is proportional to its corresponding read count and inversely proportional to an unknown effective length. Rather than fixing these effective lengths a priori, we treat their reciprocals as unknown proportionality constants. Specifically, we define:


(6) \begin{eqnarray*}{TRef}_{est}\approx{R}_{Ref}\cdotp p;\end{eqnarray*}

(7) \begin{eqnarray*}{TA}_{est}\approx{R}_A\cdotp w;\end{eqnarray*}

(8) \begin{eqnarray*} {TB}_{est}\approx{R}_B\cdotp y.\end{eqnarray*}

In an ideal case, the constants of proportionality for each path (*p*, *w*, and *y*) would be equal to its corresponding effective length: $1/{l}_A$, $1/{l}_B$, and $1/{l}_{Ref}$. Using this information, it is possible to rewrite Equation 5 as follows:


(9) \begin{eqnarray*}{R}_{Ref}\cdotp p={R}_A\cdotp w+{R}_B\cdotp y\to{R}_{Ref}={R}_A\cdotp \frac{w}{p}+{R}_B\cdotp \frac{y}{p}\end{eqnarray*}

where $u=\frac{w}{p}$ and $v=\frac{y}{p}$ as the scaling coefficients for ${Path}_A$ and ${Path}_B$ relative to the reference path, respectively. In an ideal case (uniform coverage), these would be equal to the ratios of the classical effective lengths: $u={l}_{Ref}/{l}_A$and $v={l}_{Ref}/{l}_B$. By estimating *u* and *v* from the data, EPBAM learns coverage-corrected effective-length ratios that account for locus-specific biases.

In this case, ${R}_{Ref}$, ${R}_A$, and ${R}_B$ are known and *u* and *v* are the unknowns. It is possible to solve for *u* and *v* using a least squares approach:


(10) \begin{eqnarray*}\underset{x\ge 0}{\mathit{\min}}\left\Vert Ax-b\right\Vert \end{eqnarray*}

where:

-
$A=\left[{R}_A\kern0.5em {R}_B\right]$ is a matrix ($A\in{\mathbb{R}}^{n\times 2}$) where *n* is the number of samples.
-
$b={R}_{Ref}$ is a ($b\in{\mathbb{R}}^{n\times 1}$).
-
$x=\left[\begin{array}{c}u\\{}v\end{array}\right]$ is a ($x\in{\mathbb{R}}^{2\times 1}$) vector that represents the scaling coefficients for ${Path}_A$ and ${Path}_B$, respectively.

Note that Equation 10 can be solved directly using a non-negative least squares algorithm. Nevertheless, in some scenarios—particularly with few samples—the estimation can be unstable, as the system is weakly constrained by the observed counts alone. To address this, EPBAM incorporates a penalized non-negative least squares (NNLS) formulation: a penalty term *λ* encourages the estimated coefficients to remain close to their classical effective-length ratios. When *λ* = 0, the solution is fully data-driven; as *λ* increases, the solution converges to the classical effective-length assumption. The penalty weight is set adaptively: it is proportional to *λ* times the residual deviance of the unregularized non-negative least-squares fit, divided by the squared distance between that solution and the classical effective-length ratios, and the square root of this quantity is used as the weight of the penalty rows appended to the system. This scaling makes the effective strength of the penalty independent of the units of the counts and of how far the unregularized solution already lies from the classical assumption. The default is *λ* = 0.1 (see Supplementary Text S3 for the full formulation).

For each event, the scaling coefficients are estimated jointly from all samples in the experiment in a single penalized non-negative least-squares fit, not separately per condition or per contrast, so that a single pair of coefficients applies to the whole dataset for that event. They are re-estimated within every bootstrap replicate, so that their estimation uncertainty is propagated into the Ψ and ΔΨ distributions. Because the fit pools both conditions, events with strong differential usage yield coefficients that represent a compromise between them. Conversely, the coefficients are only identifiable when Ψ varies across samples, and the penalty is what stabilizes them when it does not. A sensitivity analysis over λ, sequencing depth, replicate number, and the magnitude of the true ΔΨ is reported in Supplementary Text S9 and Supplementary Table S6. Further, an ablation analysis in which the estimated coefficients are replaced by the conventional effective-length ratios, run on the SIRV under the three annotation conditions, is reported in Supplementary Text S11 and Supplementary Table S9.

Once *u* and *v* have been estimated, the Ψ value for each sample is computed as:


(11) \begin{eqnarray*}{\Psi}_{EPBAM}^{\ast }=\frac{uR_A}{uR_A+{vR}_B}\end{eqnarray*}

where ${R}_A$ and ${R}_B$ are the read counts for Path A and Path B, respectively. This formulation replaces the classical effective lengths with the data-driven scaling coefficients, yielding a coverage-corrected Ψ estimate.

#### Bootstrap resampling

For each detected event, EPBAM performs bootstrap resampling with replacement at the read level, obtaining new estimates of read counts in each path. Then, for each bootstrap replicate, EPBAM recomputes the estimation of Ψ using the same length-normalized formulation described above. Repeating this procedure across many replicates yields thousands of estimations of Ψ for each event and sample that are used to estimate the distribution of the ΔΨ.

This strategy is conceptually similar to that implemented in EPST, with a key difference in the source of bootstrap variability. In EPST, bootstrap replicates are directly provided by the pseudo-aligner (e.g. Salmon or Kallisto), so the method only needs to compute Ψ for each bootstrap sample returned by the quantifier (Fig. 1, panel 4).

#### Statistical analysis

Once the bootstrap distributions of Ψ have been obtained for each sample, a second-level resampling is performed. For each contrast, samples are partitioned into classes according to the contrast, and the pooled set of bootstrap replicates of all samples within a class is formed. For each bootstrap iteration, one value is drawn with replacement from the pooled set of the corresponding class for every sample position, and the contrast of interest (e.g. ΔΨ between conditions) is computed using the specified design and contrast matrices. Drawing from the pooled set is equivalent to selecting a sample at random within the class and then one of its bootstrap replicates, so that both read-sampling and between-replicate variability are propagated into the resulting distribution of ΔΨ for each event and each contrast.

From this empirical ΔΨ distribution, a *P*-value is obtained for the null hypothesis |ΔΨ| < θ, where θ is a user-defined threshold (default θ = 0). The *P*-value is computed using the relationship between confidence intervals and hypothesis testing: it corresponds to the smallest significance level *α* at which the (1 − *α*) confidence interval for ΔΨ excludes the null region [−θ, θ] (Supplementary Fig. S11). Further details are provided in Supplementary Text S4. Finally, EPBAM provides FDR correction using the *q*-value R package [45]. The calibration of the resulting *P*-values and *q*-values under a true null hypothesis is characterized in Supplementary Text S7 (Supplementary Table S4 and Supplementary Figs S12 and S13).

### Independent experimental validation: SIRV data

We used Lexogen’s SIRVs [27] as a standardized, externally validated ground truth for benchmarking. SIRVs mimic endogenous transcripts with known composition, providing defined Ψ and ΔΨ values. Our study analysed 60 publicly available RNA-seq samples (ArrayExpress E-MTAB-8461) containing SIRV Set 1 Control Mix. The samples were grouped into three sets (Mix E0, E1, and E2, 20 samples each). Reads were aligned to the human genome (v38, EnsEMBL 90) extended with the SIRV genome. We focused on AS events in the seven SIRV genes, which exhibit both canonical and complex splicing patterns. Lexogen provided three annotation types: complete annotation (CA), incomplete annotation (IA), and overloaded annotation (OA), enabling assessment of annotation quality impact. SIRV Ψ calculation details are in Supplementary Text S3.

The corresponding SIRV sequences are accessible through Lexogen’s website and the National Center for Biotechnology Information (NCBI) Nucleotide Database (accession SRR3497201). Detailed experimental protocols, including cell lines, RNA extraction, and library preparation, are described by Gerbracht *et al*. [46].

### Simulated data generation

We used a simulated dataset to evaluate tool accuracy, originally created by SUPPA2 [20] and later extended by EPST [15]. The dataset features genes with two isoforms containing either CE or alternative splice site (A5/A3) events, where positive events are defined as those with ΔΨ >0.2. It comprises 554 CE and 636 A5/A3 events across two conditions with three replicates, tested at various sequencing depths (1–60 M reads).

Briefly, transcript abundances were taken from the quantification of three real control samples (GEO accession GSE59335) against the RefSeq transcriptome, and differential splicing was introduced by exchanging the abundances of the two transcripts of each selected gene. Positive events were those for which the resulting change in relative abundance exceeded 0.2. An equal number of negative events was selected without exchanging abundances, so that they carry no simulated change between conditions while retaining a comparable distribution of gene expression and of transcript relative abundance; their expected variability between conditions is therefore similar to the variability between biological replicates. Sequencing reads were then simulated with RNA-Seq by Expectation-Maximization (RSEM) at several depths and read lengths. The simulation pipeline and parameters are described in the original SUPPA2 publication; we used the dataset as provided by the authors.

### Real RNA-seq data with PCR validation

We reanalysed a previously published RNA-seq dataset from triple-negative breast cancer cell lines treated with the CK2 inhibitor CX-4945, known to affect splicing [47]. The original study details the experimental protocol. Briefly, MDA-MB-231, MDA-MB-468, and SUM149 cells were treated with either 1 μM CX-4945 or vehicle (dimethyl sulfoxide—DMSO—) for 12 h, and total RNA was extracted using the RNeasy Mini Kit (Qiagen). Libraries were prepared and sequenced on the Illumina HiSeq2000 platform with paired-end 100 bp reads in a strand-specific design. Five biological replicates were included per cell line and condition, giving 30 RNA-Seq samples in total; the data are deposited as GEO series GSE104973, the RNA-seq subseries of the SuperSeries GSE104974, whose remaining 30 samples correspond to the junction-array subseries GSE104610 and were not used here. Each cell line was analysed as a separate five-versus-five contrast. A subset of AS events was validated via end-point PCR. We re-analysed these data with all benchmarked tools, aligning sequences to human genome v38 (Ensembl 113), to compare predictions against PCR-confirmed outcomes. The data are available at Gene Expression Omnibus under accession number GSE104974.

### Processing and execution of alternative splicing tools

We applied rMATS, SUPPA2, MAJIQ, EPST, Shiba, LeafCutter, and EPBAM to the SIRV data, the simulated dataset, and the real RNA-seq datasets. For alignment-based methods (rMATS, MAJIQ, EPBAM, Shiba, and LeafCutter), we used the splice-aware aligner STAR (parameter settings are provided in Supplementary Text S6) with the same genome references employed elsewhere in this study: the SIRV-extended human reference for SIRV experiments and the human genome v38 for simulated and real data. For Shiba, STAR-aligned reads were further processed with a reference-guided transcript assembly step (StringTie2), and exon–exon and exon–intron junction reads were counted with RegTools and featureCounts, respectively. For LeafCutter, STAR-aligned reads were processed with RegTools to extract split-read junctions. The following software versions were used: rMATS v4.3.0, SUPPA2 v2.3, MAJIQ v2.5.9 + VOILA v2.5.9, STAR v2.7.10a, Kallisto v0.44.0, EventPointer v3.20 (Bioconductor release 3.23), Shiba v0.8.2, and LeafCutter v0.2.7, StringTie2 v3.0.3, RegTools v1.0.0, and featureCounts v2.1.1. Runtime, central processing unit (CPU) efficiency, and peak memory for each tool, measured on the CX-4945 dataset, are reported in Supplementary Text S8 and Supplementary Table S5. Thresholds were not harmonized across tools: each method was run under its own recommended calling criterion, because the underlying statistics—a posterior probability for MAJIQ, an empirical *P*-value for SUPPA2, and a bootstrap *P*-value for EventPointer—are not commensurable. All thresholds, non-default parameters and annotation files are reported in full in Supplementary Table S3, and the sensitivity of our conclusions to this choice is characterized in Supplementary Text S10. Unless stated otherwise, cross-tool comparisons use nominal, uncorrected *P*-values at .05 cut-off; no multiple-testing correction was applied by us. Two exceptions apply: MAJIQ was not filtered on significance and was instead used with its default effect-size threshold of 0.20, and SUPPA2 was run with gene-level *P*-value correction enabled, so its *P*-values are already adjusted.

For isoform-based methods (SUPPA2 and EPST), we used Kallisto as a pseudo-aligner with the corresponding reference transcriptomes: the SIRV reference transcriptome for SIRV data and the Ensembl 113 cDNA transcriptome (Homo_sapiens.GRCh38.cdna.all) for the real dataset, and the RefSeq hg19 transcriptome for the simulated dataset.

### Events matching

To enable a fair comparison between methods, it is necessary to establish a systematic procedure for matching events across tools. Because each software package adopts its own event definitions, labels, and underlying representations, direct one-to-one correspondence is not guaranteed. In addition to matching events between computational methods, we also require consistent mapping to the events defined in the simulated data, the SIRV controls and those experimentally validated by PCR, so that performance can be assessed against external ground truth data.

#### Locus-level detection in SIRV

Splice junctions were derived from the complete SIRV annotation, which gives 114 distinct junctions. A junction was called constitutive when all the transcripts whose genomic extent spans it use it, and alternative otherwise. This gives 27 constitutive and 87 alternative junctions.

For each tool and condition, we read the coordinates of the splice junctions defining every event (or cluster of introns for LeafCutter) reported in SIRV genes, summarized as the genomic interval they delimit, and compared these intervals with the 87 alternative junctions.

#### Matching of splicing events with SIRV

Briefly, for every event reported by EPST, EPBAM, rMATS, SUPPA2, Shiba, and MAJIQ+VOILA, we identified the genomic segments that would correspond to Path A, Path B, and the reference path depicted in Supplementary Fig. S2. For each path, the junctions and exons that define it were overlapped with the exon structure of the 69 SIRV transcripts (findSpliceOverlaps, GenomicAlignments), and we evaluated whether at least one transcript was compatible with the structure defined by the path. The complete SIRV annotation was used as the reference in the three conditions, since the incomplete and OAs are what was supplied to each tool, not what is present in the sample (Supplementary Fig. S4 and Supplementary Fig. S5). More details are provided in Supplementary Text S2.

An event was considered matched when both paths had at least one compatible SIRV transcript, and counted as a false event (FE) otherwise, meaning that their structures do not correspond to any splicing choice among the isoforms present in the genome.

Accordingly, for each tool, the reported events were partitioned into true events (TE), those matched to SIRV, and FE, those not matched. Because this partition is defined over the events that each tool reports rather than over a common catalogue, the associated FDR = FE/(TE + FE) is a precision conditional on each tool’s own set of calls.

As LeafCutter does not define events, it was matched at the intron level. Within each cluster containing at least two introns, the transcripts supporting a given intron are those that contain it exactly, and the alternative transcripts are those using any other intron of the same cluster; introns for which either group was empty were discarded.

#### Cross-tool event agreement in SIRV

Once the corresponding Path A and Path B had been defined, we required that the genomic positions of the paths from events reported by different tools overlap. This overlap was assessed using the findOverlaps function from the GenomicAlignments R package [48]. Because the labelling of the two alternative paths is arbitrary, we also checked whether Path A from one tool matched Path B from another tool and vice versa. Only the events matched to SIRV were considered and LeafCutter was excluded from this comparison, as it does not define events.

#### Matching with simulated data

To match the events reported by each tool with the events of the simulated dataset, we followed the same strategy used for the SIRV data. Once the events of a given tool (but LeafCutter) had been expressed as Path A and Path B, their genomic coordinates were compared with the coordinates that define the simulated events themselves, which are provided as SUPPA event identifiers. LeafCutter was handled in the opposite direction, since its intron clusters have no equivalent path-pair structure: each simulated event was instead written as the set of introns that define it—the two alternative junctions, plus the implicit skipping junction in cassette-exon events—and the event was considered detected when a single LeafCutter cluster contained all of them, the cluster-level *P*-value being assigned to that event. Further details are provided in Supplementary Texts S2 and S5.

#### Ψ and ΔΨ accuracy in SIRV

Ψ and ΔΨ were taken as defined by each tool. For MAJIQ+VOILA, a specific Ψ and ΔΨ were derived to make the comparison possible (Supplementary Text S3). ΔΨ was computed for the three contrasts, Mix1 versus Mix2, Mix1 versus Mix3, and Mix2 versus Mix3; details on how the *P*-values were obtained are given in Supplementary Text S4. The SIRV ground truth Ψ and ΔΨ were derived from the known abundance ratios of the transcripts in each mix (Supplementary Text S3). Estimated and reference values were compared in the three annotation conditions using the Pearson correlation coefficient and the root mean squared error; for ΔΨ, events called significant (*P* < .001) were separated from those that were not.

#### Differential-event classification in SIRV

An event was taken to change when |ΔΨ| in the SIRV design exceeded 0.2, the same criterion used with the simulated data, and to be called by a tool when its raw *P*-value was below .001. The analysis was restricted to the events already matched to SIRV. Sensitivity, specificity, precision, and FDR were computed from the resulting confusion matrix, and the area under the receiver operating characteristic (ROC) curve by sweeping the statistic of each tool.

## Results

### Simulated data with low-complexity events reveals comparable performance across tools

The simulated dataset includes genes with annotated splicing patterns containing only two isoforms with a SE or alternative splice site (A5/A3) event. Since the simulated events are generated according to the annotated transcriptome, annotation-based methods are expected to have an inherent advantage. Indeed, annotation-based tools (EPST and SUPPA2, shown with triangles in Fig. 2) exhibit the strongest performance. Among *de novo* tools, rMATS, Shiba, and EPBAM discover a similar number of events (rMATS slightly more), but EPBAM and Shiba achieve a substantially lower FDR [FDR = FP/(TP + FP), below 2% even at very shallow sequencing depths]. MAJIQ and LeafCutter detect fewer events overall but with no false positives. As expected, all methods improve with increasing sequencing depth.

**Figure 2:**
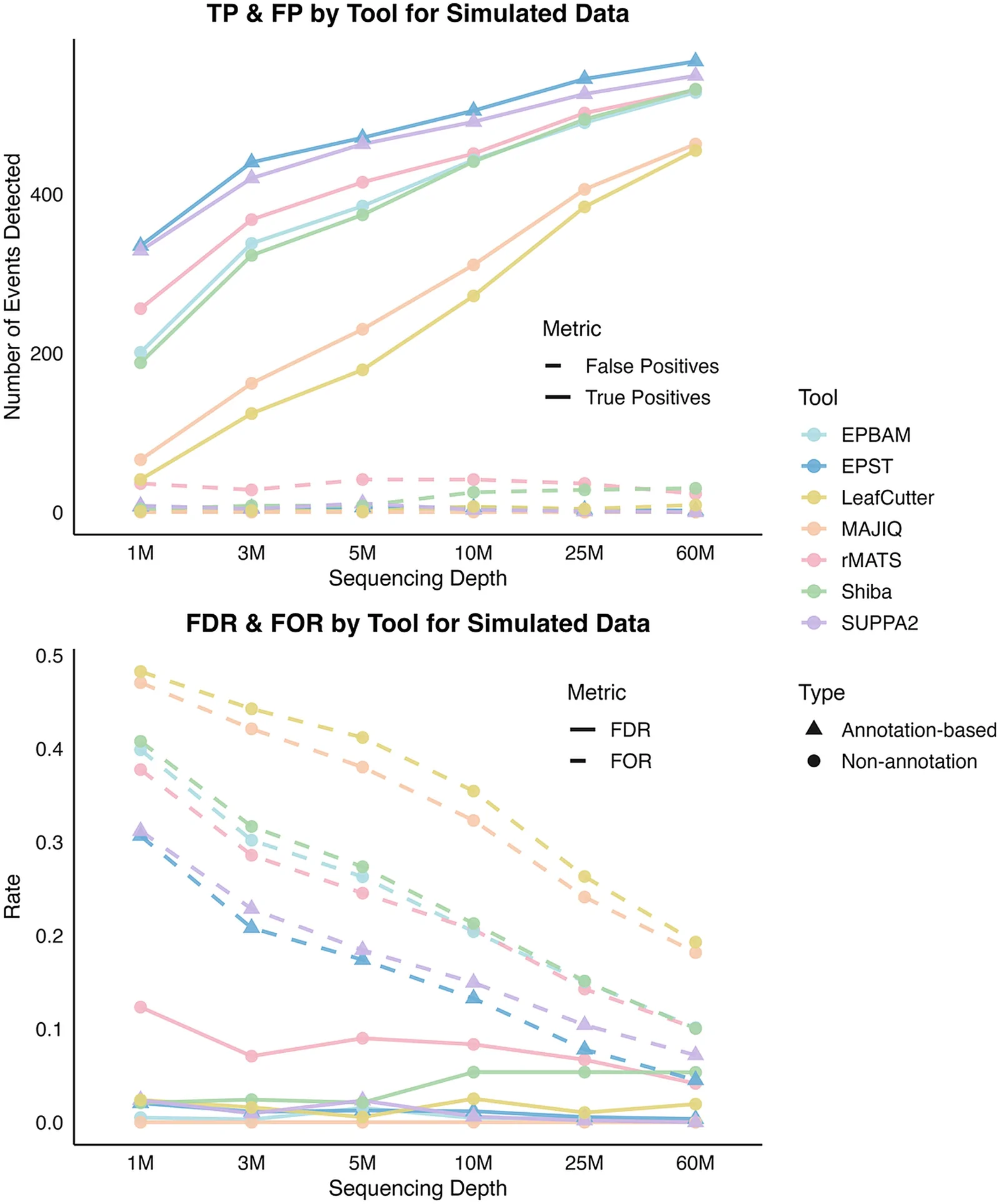
Performance of AS detection tools on simulated datasets across varying sequencing depths; top: number of true positive (TP, solid lines) and false positive (FP, dashed lines) events detected by each tool—EPBAM (light blue), EPST (dark blue), LeafCutter (yellow), MAJIQ (orange), rMATS (pink), Shiba (green), and SUPPA2 (purple)—as a function of sequencing depth (1–60 M reads); bottom: FDR and FOR for each tool at different sequencing depths (lower is better); triangles indicate the annotation-based tools (EPST and SUPPA2) and circles the remaining tools, which do not rely on the annotation to define events.

We also report the False Omission Rate [FOR = FN/(FN + TN)] for this simulation of 594 events. MAJIQ and LeafCutter, detecting fewer events, exhibit the largest FOR. At sequencing depths of 10 M reads or more—below the typical depth of current RNA-seq experiments—all methods achieve FOR values between ~10% and 20%, reflecting a non-trivial rate of missed events that represents a trade-off with the stringency of event calling.

We note that this simulation is limited to simple canonical events in two-isoform genes and does not capture the complexity of real splicing landscapes, where genes may harbour multiple isoforms and complex event architectures. We therefore regard the SIRV analysis below as the primary benchmarking dataset.

### The SIRV dataset provides a realistic ground truth for comprehensive benchmarking

While the simulated data provide a useful baseline, it is limited to annotated, canonical, and simple events with fabricated sequencing characteristics. The Lexogen SIRV data addresses these limitations: it includes both simple and complex events (Supplementary Tables S1 and S2), uses real sequencing data rather than simulations, and provides exact concentrations that serve as ground truth for quantitative comparison. Critically, Lexogen provides three annotation tiers: CA, containing all expressed isoforms; IA, omitting some expressed isoforms to simulate an incomplete reference; and OA, including additional non-expressed isoforms to simulate an over-annotated reference. This enables systematic assessment of how annotation quality affects tool performance—a dimension rarely evaluated in benchmarking studies.

Throughout this section, we refer to SUPPA2 and EPST as ‘annotation-based tools’ (relying solely on annotation for event detection) and to EPBAM, rMATS, Shiba, LeafCutter, and MAJIQ as ‘de novo tools’ (capable of detecting novel unannotated events).

### Annotation-derived event structure determines sensitivity to annotation quality

We assessed whether each tool defines at least one event at every splice junction present in the SIRV transcripts, independently of how it delimits that event (Methods). The CA defines 87 alternative splice junctions. The IA retains 46 of them, while the OA contains all 87 plus 24 junctions that belong to non-expressed isoforms and therefore have no read support in the samples (Supplementary Table S2). A junction was counted as addressed when at least one reported event spanned it, using the complete event catalogue of each tool before any matching against SIRV. Coverage is expressed over the same 87 junctions in the three conditions. For LeafCutter, we consider a junction counted if it is considered in at least one intron cluster. Because this comparison uses each tool’s own event catalogue, it is independent of the matching framework, and therefore free of the biases that the mapping may introduce in the event-level comparisons that follow.

Under CA, the annotation-based tools address the largest number of junctions: EPST 84 (96.6%) and SUPPA2 79 (90.8%), followed by EPBAM with 77 (88.5%) and Shiba with 76 (87.4%); rMATS addresses 58 (66.7%) (Table 2). The union of the seven tools covers all 87 junctions, so the reference set is attainable, and the gaps are specific to each method.

**Table 2:** Locus event detection by each tool in the CA, the IA, and the overload annotation (OA).

	Tool	Junctions	Covered (*/87)	Coverage (/87) (%)	False
**CA**	rMATS	87	58	66.7	–
	EPBAM	87	77	88.5	–
	MAJIQ	87	63	72.4	–
	Shiba	87	76	87.4	–
	EPST	87	84	96.6	–
	SUPPA2	87	79	90.8	–
	LeafCutter	87	70	80.5	–
**IA**	rMATS	46	40	46	–
	EPBAM	46	73	83.9	–
	MAJIQ	46	57	65.5	–
	Shiba	46	69	79.3	–
	EPST	46	46	52.9	–
	SUPPA2	46	45	51.7	–
	LeafCutter	46	70	80.5	–
**OA**	rMATS	111	68	78.2	14
	EPBAM	111	76	87.4	13
	MAJIQ	111	63	72.4	10
	Shiba	111	83	95.4	20
	EPST	111	83	95.4	18
	SUPPA2	111	84	96.6	20
	LeafCutter	111	73	83.9	17

Junctions: Number of alternative splice junctions present in the annotation used in each corresponding condition. Covered and coverage (%): Number and percentage of the 87 real alternative junctions covered. False: Number of the 24 annotation-only junctions covered by the tool; it is zero in CA and IA because those annotations contain no such junctions.

Under IA, EPST and SUPPA2 fall to 46 (52.9%) and 45 (51.7%), respectively, since the junctions removed from the annotation cannot be recovered from the reads, i.e. in the IA, EPST and SUPPA2 are limited to the 46 splice junctions. The tools that derive event structure from the alignments are less affected and define events at junctions absent from the reference: EPBAM addresses 73 junctions (83.9%) and becomes the tool with the widest coverage, Shiba with 69 (79.3%) and MAJIQ with 57 (65.5%). LeafCutter returns the same 70 junctions as under CA, as expected from a method that builds intron clusters from split reads alone.

Under OA, coverage of the real junctions recovers for all tools, but every method also defines events at junctions that are only present in the annotation. SUPPA2 addresses 20 of these 24 junctions and EPST 18; among the remaining tools the values range from 10 for MAJIQ and 13 for EPBAM to 20 for Shiba (Table 2).

Finally, rMATS is the most reference-dependent of the splice-aware tools, losing 20.7 percentage points between CA and IA against 4.6 for EPBAM, since it derives its event templates from the annotation. EPBAM addresses 77, 73, and 76 junctions across the three conditions, the most stable loci detection of all the tools evaluated. Supplementary File S2 gives the number of events each tool builds on every junction.

### Tools report different numbers of events and rarely agree on their structure

The rows in Table 3 labelled ‘Total events’, ‘SIRV’, and ‘FE’ display the number of discovered events for each tool, how many matched the SIRV ground truth and how many did not. The row labelled ‘***’ indicates the number of events statistically significant at *P* < .001. For LeafCutter, instead of events, we report the number of different introns considered within each intron cluster. Under the CA condition, annotation-based tools achieve the highest accuracy: EPST identifies 63 events (0 false) and SUPPA2 identifies 50 events (0 false), as expected when all events are present in the reference. Among *de novo* tools, EPBAM detects 51 events with only 3 false positives (FDR = 5.9%), while MAJIQ detects 66 events with 9 false positives (FDR = 13.6%). rMATS detects 24 events with 2 false positives (FDR = 8.3%). Shiba detects 81 events with 21 false positives (FDR = 25.9%), and LeafCutter reports 68 introns with 2 false positives (FDR = 2.9%).

**Table 3:** Number of AS events detected for each AS tool under three different annotation scenarios: complete, incomplete, and overload.

		CA			IA				OA		
	#Events	Total events	SIRV	FE	Total events	SIRV	FE	DN	Total events	SIRV	FE
**rMATS**	*Total*	24	22	2	20	19	1	6	47	32	15
	***	21	21	0	18	18	0	5	37	30	7
**EPBAM**	*Total*	51	48	3	47	45	2	20	51	48	3
	***	40	38	2	40	39	1	17	40	38	2
**MAJIQ + VOILA**	*Total*	66	57	9	53	44	9	22	70	59	11
	***	54	46	8	49	40	9	18	55	47	8
**EPST**	*Total*	63	63	0	38	38	0	–	75	75	0
	***	61	61	0	38	38	0	–	63	63	0
**SUPPA2**	*Total*	50	50	0	24	24	0	–	54	54	0
	***	48	48	0	23	23	0	–	49	49	0
**Shiba**	*Total*	81	60	21	57	50	7	22	93	53	40
	***	60	53	7	51	46	5	19	67	49	18
**LeafCutter**	*Total introns*	68	66	2	72	66	6	45	78	68	10
	***	68	66	2	72	66	6	45	75	68	7

The total number of detected events includes novel (DN) events in the IA condition for tools that support this capability. The triple asterisk indicates that the corresponding *P*-value is below .001. FE indicates false events that do not match against the SIRV ground truth. For LeafCutter, instead of total events, the table reports the number of introns detected within all intron clusters. FE counts, and any rate derived from them, are conditional on the matching procedure described in methods and in Supplementary Text S2.

The IA condition is particularly informative, as it tests the ability of *de novo* tools to discover unannotated events (labelled as ‘DN’ in Table 3). EPBAM detects 20 novel events (not present in the IA), compared to 6 for rMATS, 22 for MAJIQ, and 22 for Shiba. The FDR associated with these detections is 17.0% for MAJIQ [9/(9 + 44)], 12.3% for Shiba [7/(7 + 50)], 5.0% for rMATS [1/(1 + 19)], and 4.3% for EPBAM [2/(2 + 45)]. Annotation-based tools (EPST and SUPPA2) cannot detect novel events by design. LeafCutter reports 45 introns with a FDR of 8.3% [6/(6 + 66)].

EPBAM’s event detection is stable across annotation conditions: 47 total events under IA (2 false), 51 under CA (3 false), and 51 under OA (3 false), and LeafCutter varies within a comparable range (68, 72, and 78) introns detected. In contrast, rMATS detected 24 events under CA but 47 under OA (with 15 false positives), SUPPA2 detected 50 events under CA but only 24 under IA. This stability reflects EPBAM’s approach of constructing the splice graph primarily from aligned reads, with annotations serving as supplementary rather than deterministic input. On the other hand, Shiba shows the largest number of unmatched events (40 out of 93 under OA), and 31 of them arise from the same effect: Shiba merges the annotation with the StringTie2 assembly before deriving its splice graph, so the non-expressed isoforms also become events. Note that these counts are conditional on the matching framework adopted here: each tool has its own event universe, so the false-event figures are not universal FDRs and are not strictly comparable across tools whose event definition departs from the path-pair representation (Methods; Supplementary Text S2).

Figure 3 shows the overlap of detected events across tools under CA. LeafCutter is not included, as it does not define events. Five events are detected by all six tools. The strongest overlap occurs between EPBAM and EPST (19 events), which share the same event definition framework. All events detected by rMATS are also detected by SUPPA2, consistent with their shared focus on canonical patterns. MAJIQ+VOILA events show limited overlap with other tools (10 events are detected by MAJIQ alone), reflecting its distinct LSV-based event definition. Shiba contributes 13 tool-specific events and overlaps mainly with SUPPA2 and MAJIQ. OA and IA comparisons are provided in Supplementary Figs S6 and S7.

**Figure 3:**
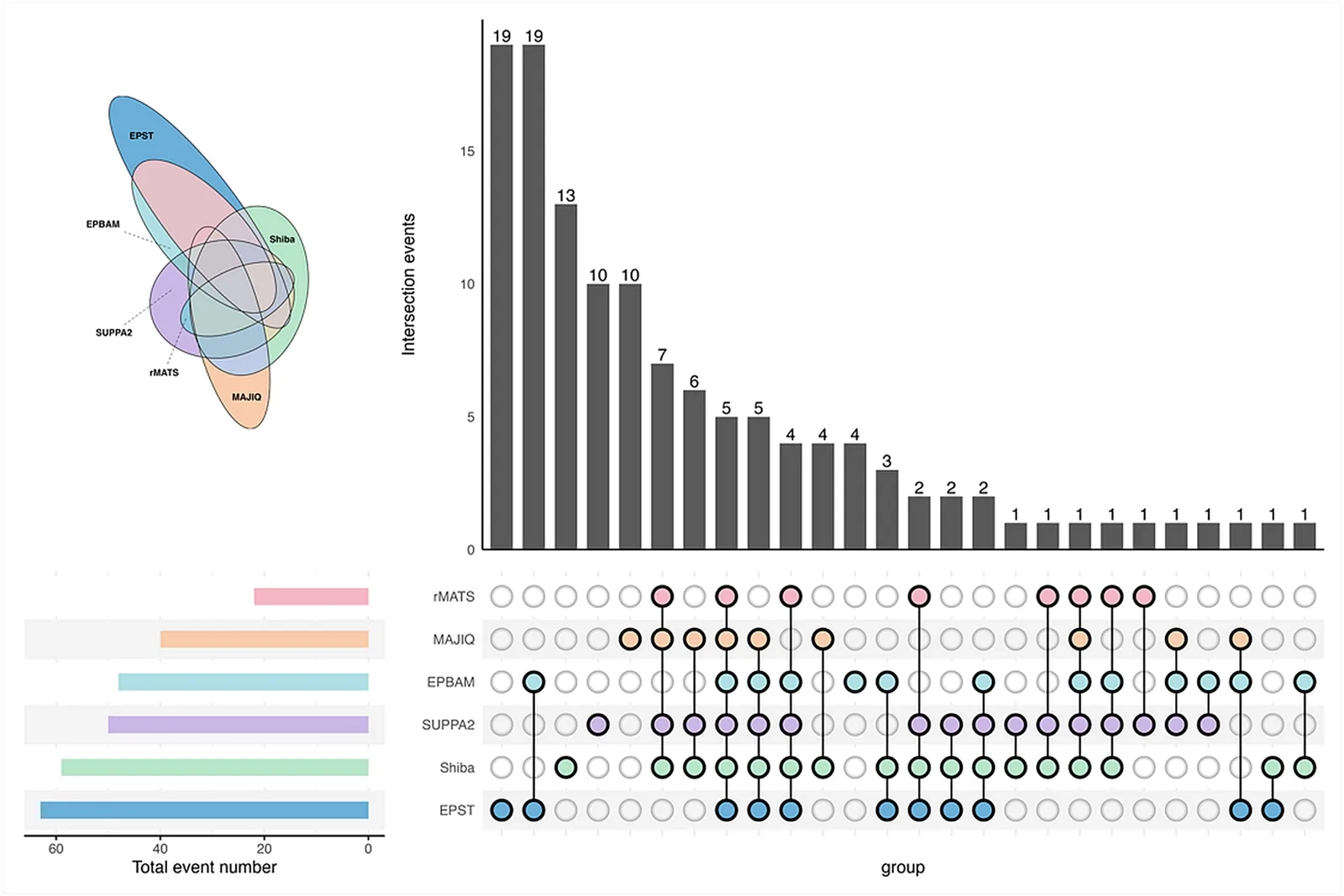
Overlap of detected events across tools under CA; (a) Venn diagram showing pairwise and higher-order overlaps; (b) UpSet plot showing the exact sizes of all intersection sets; both representations are provided as they highlight complementary aspects of inter-tool agreement.

### Annotation-based tools achieve higher Ψ accuracy; ΔΨ estimates are more reliable across all tools

The accuracy of Ψ estimation was assessed using SIRV reference data across the three annotation conditions (Fig. 4 and Table 4). Pearson (ρ) correlation was computed to compare predicted Ψ with the known SIRV value for each event.

**Figure 4:**
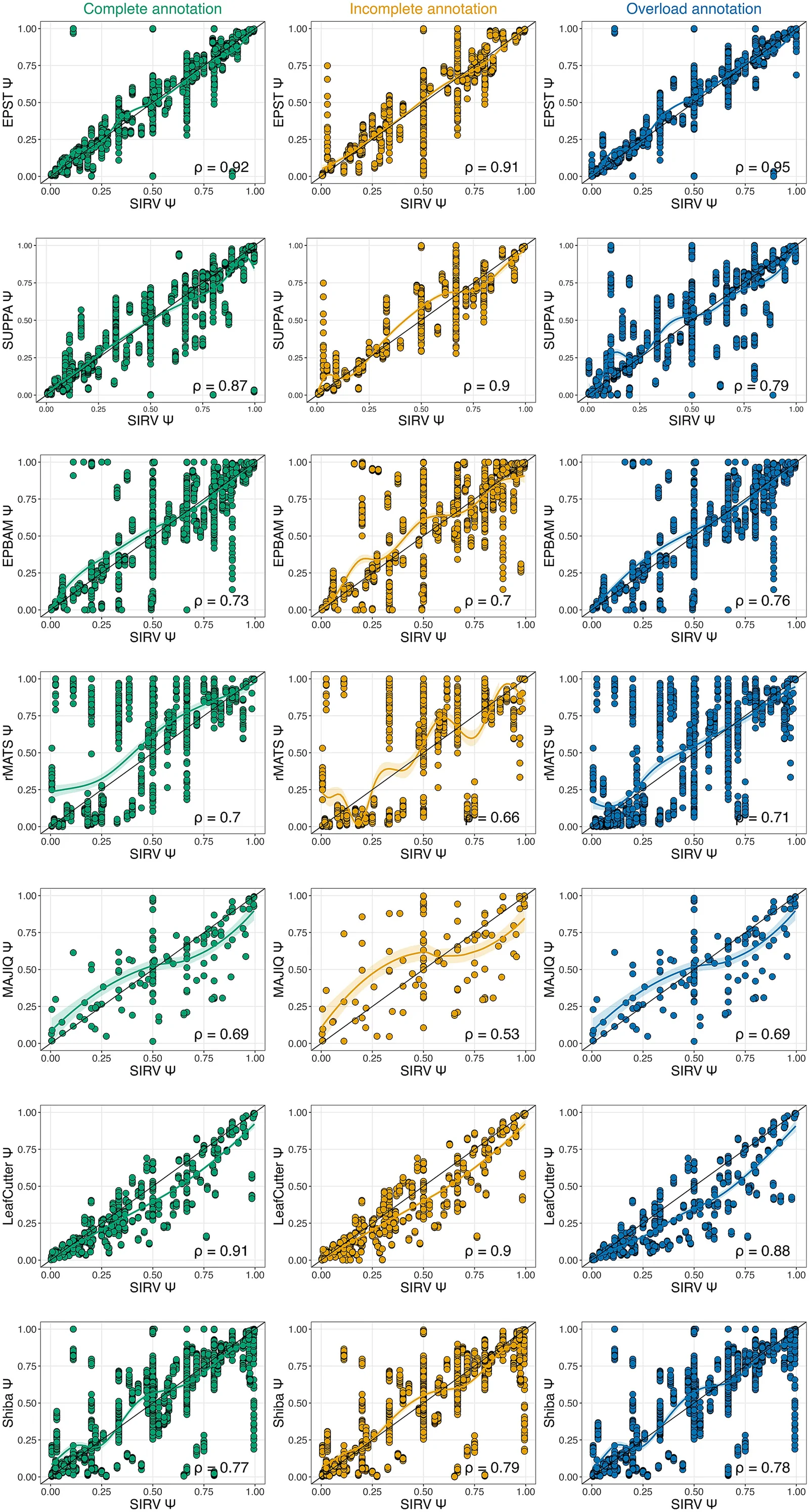
Comparison of AS tools in Ψ estimation across different annotation conditions; scatter plots illustrate the correlation between predicted Ψ values (*y*-axis) from each tool and the SIRV reference Ψ values (*x*-axis) under three annotation scenarios: complete, incomplete, and overload; each point represents a Ψ value for a specific sample under one of these conditions; for LeafCutter, an intron Ψ instead of an event Ψ; the diagonal line represents the identity line where predicted Ψ values would exactly match reference values; Pearson correlation coefficients (ρ) are indicated for each tool and annotation condition, reflecting the accuracy of Ψ estimations.

**Table 4:** Performance metrics of AS tools in ΔΨ and Ψ estimation across complete, incomplete, and overload annotation conditions.

		CA		IA		OA	
		$\boldsymbol{\mathrm{\rho}} \left(\boldsymbol{\Psi}, {\boldsymbol{\Psi}}_{SIRV}\right)$	$\boldsymbol{\mathrm{\rho}} \left(\boldsymbol{\Delta} \boldsymbol{\Psi}, \boldsymbol{\Delta} {\boldsymbol{\Psi}}_{SIRV}\right)$	$\boldsymbol{\mathrm{\rho}} \left(\boldsymbol{\Psi}, {\boldsymbol{\Psi}}_{SIRV}\right)$	$\boldsymbol{\mathrm{\rho}} \left(\boldsymbol{\Delta} \boldsymbol{\Psi}, \boldsymbol{\Delta} {\boldsymbol{\Psi}}_{SIRV}\right)$	$\boldsymbol{\mathrm{\rho}} \left(\boldsymbol{\Psi}, {\boldsymbol{\Psi}}_{SIRV}\right)$	$\boldsymbol{\mathrm{\rho}} \left(\boldsymbol{\Delta} \boldsymbol{\Psi}, \boldsymbol{\Delta} {\boldsymbol{\Psi}}_{SIRV}\right)$
**rMATS**	$\mathrm{\rho} \left(\right)$	0.701	0.657	0.663	0.649	0.711	0.677
	*RMSE*	0.275	0.241	0.297	0.233	0.267	0.224
**EPBAM**	$\mathrm{\rho} \left(\right)$	0.730	0.898	0.699	0.912	0.758	0.947
	*RMSE*	0.242	0.137	0.258	0.122	0.229	0.098
**MAJIQ + VOILA**	$\mathrm{\rho} \left(\right)$	0.69	0.89	0.53	0.86	0.69	0.9
	*RMSE*	0.221	0.207	0.281	0.24	0.222	0.202
**EPST**	$\mathrm{\rho} \left(\right)$	0.92	0.98	0.91	0.97	0.95	0.99
	*RMSE*	0.125	0.049	0.122	0.075	0.113	0.046
**SUPPA2**	$\mathrm{\rho} \left(\right)$	0.87	0.87	0.85	0.94	0.79	0.85
	*RMSE*	0.164	0.17	0.172	0.124	0.203	0.183
**Shiba**	$\mathrm{\rho} \left(\right)$	0.77	0.87	0.79	0.88	0.78	0.87
	*RMSE*	0.207	0.163	0.196	0.156	0.206	0.158
**LeafCutter**	$\mathrm{\rho} \left(\right)$	0.91	0.93	0.9	0.92	0.88	0.91
	*RMSE*	0.138	0.102	0.142	0.105	0.168	0.103

The table summarizes the Pearson correlation (ρ), RMSE, and for each AS tool under three different annotation scenarios: complete, incomplete, and overload. For LeafCutter, the table reports the Pearson correlation (ρ) and RMSE of the introns.

Annotation-based tools consistently outperformed *de novo* tools in Ψ estimation. EPST achieved the highest correlations across all conditions (*ρ* ≥ 0.91), and both EPST and SUPPA2 showed stable performance between CA and IA. However, the two tools responded differently to OA: EPST’s correlation improved (*ρ* = 0.95), whereas SUPPA2’s dropped to *ρ* = 0.79. This divergence reflects differences in how each tool handles non-expressed annotated isoforms: the additional transcripts in OA disrupted SUPPA2’s canonical pattern definitions but did not adversely affect EPST’s splice-graph-based quantification. These results suggest that isoform-level quantification from pseudo-aligners, which leverages information from the entire transcript, provides more accurate absolute Ψ estimates than read-level quantification restricted to the vicinity of the event.

Among *de novo* tools, Shiba achieved the highest Ψ correlations (CA *ρ* = 0.77, IA *ρ* = 0.79, OA *ρ* = 0.78; range = 0.02), followed by EPBAM (CA *ρ* = 0.73, IA *ρ* = 0.70, OA *ρ* = 0.76; range = 0.06), rMATS (CA *ρ* = 0.70, IA *ρ* = 0.66, OA *ρ* = 0.71; range = 0.05), and MAJIQ (CA *ρ* = 0.69, IA *ρ* = 0.53, OA *ρ* = 0.69; range = 0.16).

ΔΨ estimates were substantially more accurate than Ψ estimates across all tools (Fig. 5), with higher correlations and lower root mean square error (RMSE) values. This pattern suggests the presence of systematic biases in absolute Ψ quantification—arising from coverage non-uniformity, mapping ambiguities, or effective-length approximations—that are largely cancelled when computing differences between conditions. An alternative explanation—that the event-matching procedure introduces errors in absolute Ψ comparison but not in within-tool ΔΨ calculations—cannot be fully excluded, although the consistency of this pattern across all seven tools with different event definitions argues against matching artefacts as the primary explanation.

**Figure 5:**
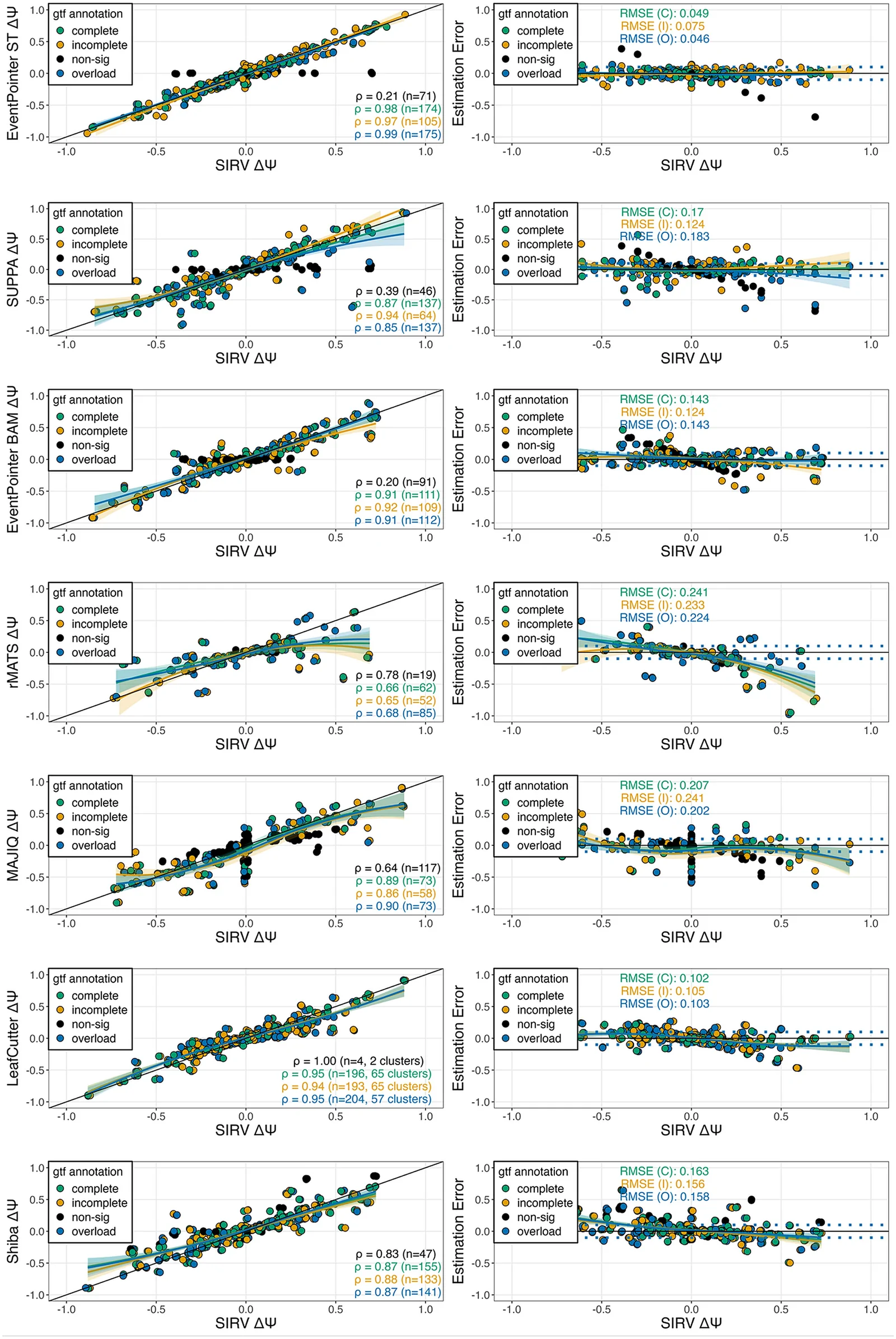
Performance of AS tools in ΔΨ estimation across varying annotation conditions; scatter plots (left) show the correlation between the predicted ΔΨ values from each tool (*y*-axis) and the SIRV ΔΨ reference values (*x*-axis) under complete, incomplete, and overload annotation conditions; each point represents a ΔΨ value for a specific splicing event under one of the annotation conditions being compared; for LeafCutter, an intron ΔΨ instead of an event ΔΨ; points are colour-coded by annotation condition; for LeafCutter the number of independent clusters is also shown, and correlations are weighted by the inverse of the number of introns per cluster.

EPST achieved the highest ΔΨ correlation and lowest RMSE among all tools (*ρ* up to 0.99 under OA, RMSE = 0.046). Among *de novo* tools, EPBAM and MAJIQ gave the highest ΔΨ correlations across the three conditions (EPBAM *ρ* = 0.90, 0.91, and 0.95; MAJIQ *ρ* = 0.89, 0.86, and 0.90), followed by Shiba (*ρ* = 0.87, 0.88, and 0.87) and rMATS (*ρ* = 0.66, 0.65, and 0.68). EPBAM also achieved the lowest RMSE (0.137, 0.122, and 0.098), followed by Shiba (0.163, 0.156, and 0.158), MAJIQ (0.207, 0.24, and 0.202), and rMATS (0.241, 0.233, and 0.224).

Finally, LeafCutter, evaluated at the intron level rather than at the event level, reported correlations with the SIRV values of 0.91, 0.90, and 0.88 for Ψ (RMSE 0.138, 0.142, and 0.168) and of 0.93, 0.92, and 0.91 for ΔΨ (RMSE 0.102, 0.105, and 0.103) under CA, IA, and OA, respectively.

#### Contribution of the coverage correction

To establish how much of EPBAM’s quantification accuracy is attributable to the coverage-corrected effective-length estimation (see Methods), we repeated the SIRV analysis with the coefficients held fixed at their conventional effective-length ratios, leaving everything else unchanged (Supplementary Text S11 and Supplementary Table S9). The correction improved both Ψ and ΔΨ under the three annotation conditions; its main effect was a reduction of bias: the mean signed error of Ψ fell from 0.087, 0.090, and 0.077 to 0.027, 0.025, and 0.018 under CA, IA, and OA. We have also examined the sensitivity to the value of λ (Supplementary Text S9 and Supplementary Table S6): within the range examined, the estimates are largely insensitive to it, as the penalty only stabilizes the coefficients for events whose Ψ varies little across samples.

### Differential event classification

We evaluated differential-event classification for each tool in each condition. An event was taken to change when |ΔΨ| in the SIRV design exceeded 0.2. Table 5 reports, for each tool and condition, the number of events matched to SIRV that were available for the comparison and the area under the ROC curve (Supplementary Fig. S8).

**Table 5:** An event was taken to change when |ΔΨ| in the SIRV design exceeded 0.2.

	Tool	*N*	AUC
CA	**rMATS**	66	0.601
	**EPBAM**	144	0.827
	**MAJIQ + VOILA**	171	0.748
	**EPST**	189	0.731
	**SUPPA2**	150	0.573
	**Shiba**	176	0.704
	**LeafCutter**	66*	0.647
IA	**rMATS**	56	0.759
	**EPBAM**	135	0.83
	**MAJIQ + VOILA**	132	0.793
	**EPST**	114	0.905
	**SUPPA2**	72	0.648
	**Shiba**	146	0.748
	**LeafCutter**	66^*^	0.687
OA	**rMATS**	96	0.710
	**EPBAM**	144	0.818
	**MAJIQ + VOILA**	177	0.767
	**EPST**	222	0.817
	**SUPPA2**	162	0.509
	**Shiba**	154	0.702
	**LeafCutter**	57*	0.551

*N* is the number of event-by-contrast observations available for each tool. It is at most three times the number of matched events reported in Table 3, one observation per event and pairwise mix contrast, and is smaller whenever a tool returned no statistic for an event in a given contrast or the SIRV design does not define a ΔΨ for it. (*) For LeafCutter, *N* is the number of clusters. AUC reports the area under the ROC curve.

EPBAM gave the highest and most stable values (0.827, 0.83, and 0.818), followed by MAJIQ+VOILA (0.748, 0.793, and 0.767). EPST was the best tool under IA (0.905) but fell to 0.731 under CA, and SUPPA2 gave the lowest values, reaching 0.509 under OA.

Finally, LeafCutter reached area under the curve (AUC) values of 0.647, 0.687, and 0.551 despite estimating ΔΨ with correlations of 0.91–0.93. Its *P*-value is computed for the intron cluster and shared by all the introns it contains, so it cannot discriminate between them.

### Performance on real biological data with PCR validation

#### Alternative splicing event definition influence in event detection and definition

We re-analysed a published RNA-seq dataset from triple-negative breast cancer cell lines treated with CX-4945, a drug that affects splicing. For each event, end-point reverse transcription PCR (RT-PCR) was performed using primers flanking the event of interest (348 RT-PCRs corresponding to 29 events). Six events with PCR bands not matching the annotation were excluded, leaving 23 evaluable events. Most validated events were selected to be simple canonical events in the original study. Table 6 shows, for each tool, whether the splicing event was detected and how it was classified. The first two columns of the table report the gene name and the type of event observed by PCR. Supplementary Fig. S14 shows, for each of the 23 evaluable events, the Ψ profile measured by RT-PCR across the 30 samples together with the estimates of the seven tools.

**Table 6:** Classification of the events by each tool.

Gene	Event type	EPBAM	EPST	rMATS	SUPPA	MAJIQ + VOILA	LeafCutter	Shiba
ACADM	CE	CE		CE	CE	CE	clu_2749	CE
C1orf43	CE		Complex (CE) Complex (MCE)	CE	CE	CE	clu_1267	CE
COPS7A	CE	CE		CE	CE	CE	clu_8548	CE
CPSF2	CE	Complex (CE) MCE		CE		CE	clu_11128	CE
CTC1	ALT3		Complex (ALT3)	ALT3		ALE	clu_14303	RI
DONSON	Complex (MCE)	MCE	Complex	MCE		MCE	clu_23021	MCE
GPBP1	CE	Alt First	Complex	CE		CE	clu_27713	CE
KDM1B	Complex (MCE)	MCE	MCE	MXE		CE	clu_30214	CE
KIF14	CE	CE		CE		CE	clu_1582	CE
KIF20B	Complex (MCE)	CE				RI	clu_4282	CE
MELK	CE	CE	CE	CE	CE	CE		CE
MRFAP1	RI							AFE
OSGIN2	CE	CE		CE		CE	clu_33795	CE
POLR3C	RI	RI	RI	RI	RI	RI		RI
PSMG1	ALT5	Complex	Complex (ALT5) Complex (CE)	CE		CE	clu_23053	CE
RNF185	Complex (MCE)	MCE	Complex	CE		MCE		MCE
SAYSD1	CE	CE	MCE	CE	CE	MCE	clu_29528	CE
TOP2B	CE		Complex	CE	CE	CE	clu_25130	CE
TOR1AIP2	Complex (MCE)	CE	Complex	MXE	CE	CE	clu_1511	MCE
UBQLN1	CE		Complex (ALT5) Complex (CE)	CE		CE	clu_34283	ALT5
USP9X	CE	CE		CE		CE	clu_35769	CE
WNK1	CE	Complex (CE)	CE	MXE		CE		CE
ZNF692	ALT3		Complex (ALT3)	ALT3	ALT3	ALT3		CE

ALT3: Alternative 3′ site, ALT5: Alternative 5′ site, ALE: Alternative last exon, AFE: Alternative first exon, CE: Cassette exon, RI: Retained intron, complex: Complex event, MCE: Multiple cassette exons, MXE: Mutually exclusive exons.

Shiba detects all 23 events, MAJIQ 22, rMATS 21, EPBAM, LeafCutter 17, EPST 15, and SUPPA2 9. Two events (SAYSD1 and TOR1AIP2) are detected by all seven tools, and every event except MRFAP1, which only Shiba detects, is detected by at least two tools (Table 6). The limited overlap is driven by SUPPA2: its empirical *P*-value is bounded and is therefore considerably more restrictive at a nominal *P* < .05 than the statistics of the other methods, so that the same nominal cut-off does not represent the same stringency across tools. Excluding SUPPA2, 10 events are common to EPBAM, EPST, rMATS, and MAJIQ. Below, we highlight different characteristics of the events and how they affect the behaviour of each method. In genes with a simple structure (MELK, POLR3C, SAYSD1, and WNK1), the tools show almost complete agreement, the exceptions being SUPPA2 in WNK1, and LeafCutter in MELK, POLR3C, and WNK1—the latter reflecting that LeafCutter resolves splicing at the level of intron clusters and does not represent intron retention as a distinct event.

Novel splicing events (KIF14, KIF20B, OSGIN2, TOR1AIP2, and USP9X) are detected only by EPBAM, rMATS, or MAJIQ, revealing the weakness of EPST and SUPPA2 in this type of scenario. The Sashimi plot (Fig. 6a) illustrates that there is an exon missing from the annotation. A second, weakly expressed exon appears only in the CX-4945-treated samples. Figure 6b shows how the EPBAM SG detects this exon, whereas the EPST SG does not.

**Figure 6:**
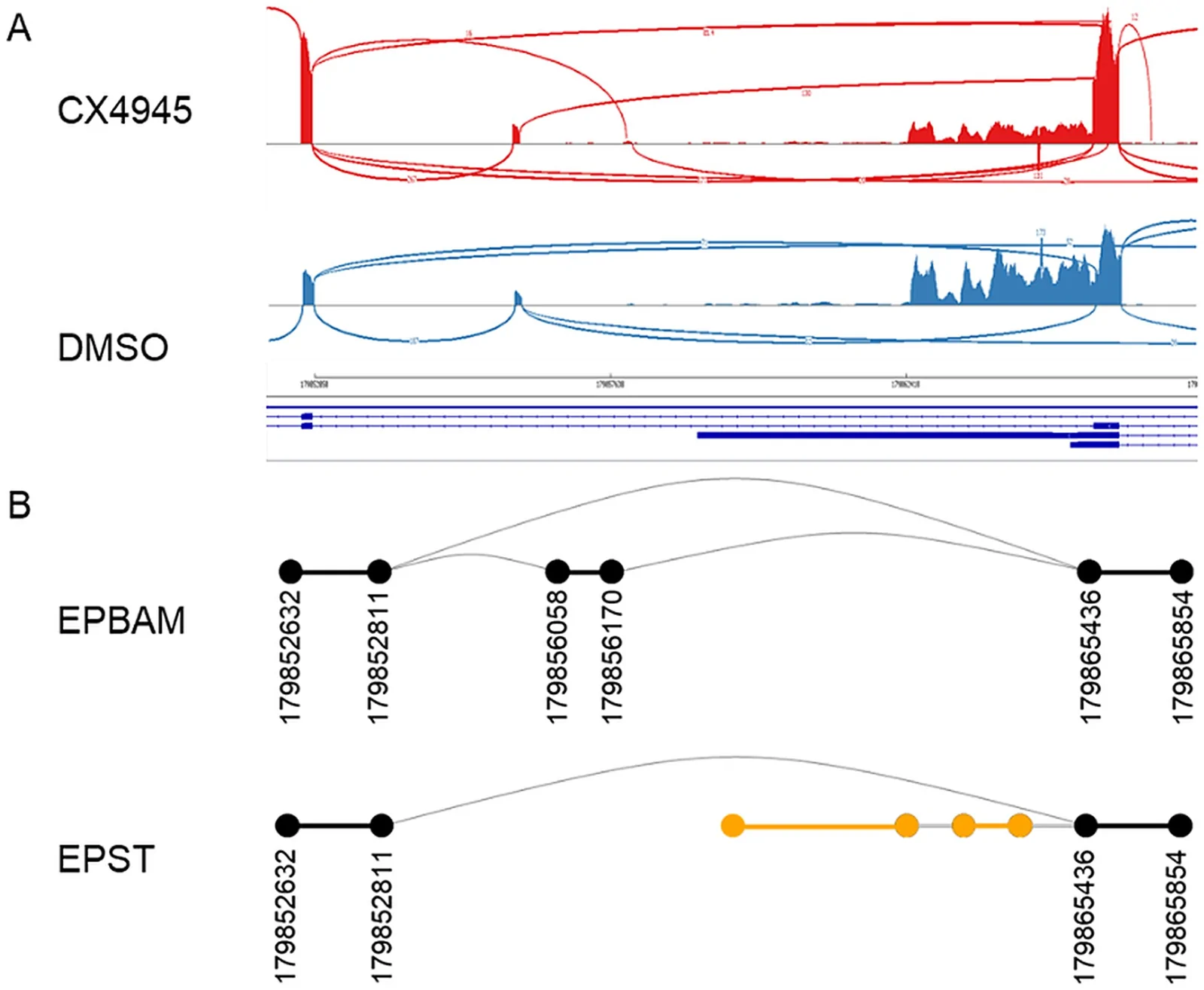
Sashimi plot, annotation, and SGs for the TOR1AIP2 gene; the sashimi plot indicates a very complex splicing pattern (much more complex that the annotation); in fact there is a non-annotated exon (and probably another one weakly expressed) and a large number of junction reads that are in turn, differential in both conditions; this complex pattern makes it difficult the comparison across the different methods.

The simplified SG in Fig. 6b illustrates how splice-aware tools can have an advantage even for annotated events. This occurs in C1orf43, GPBP1, TOP2B, and UBQLN1. In these cases, EPST classifies the events as complex (due to transcriptome complexity), whereas the other splice-aware methods classify them as canonical. These examples also highlight an important difference between EPST and SUPPA2: although both methods rely exclusively on the reference transcriptome, SUPPA2 does not consider all transcripts in the region of interest, which allows it in some cases to classify these events as canonical.

We also observed cases where the reference transcriptome structure is so complex that EPST is unable to detect the splicing event (ACADM and COPS7A). Figure 7 shows the case of ACADM: this gene has ˃40 annotated isoforms (only 10 are shown in Fig. 7a). Specifically, this splicing event is flanked by RIs that EPST includes in its SG (Fig. 7b). Because the SG is so complex, it is not possible to define an event in this region that satisfies EPST’s event definition. In contrast, the remaining methods do not consider these introns (because they are not expressed in the case of splice-aware methods and because of how SUPPA defines splicing events), resulting in a simpler SG in which the event can be detected and correctly classified.

**Figure 7:**
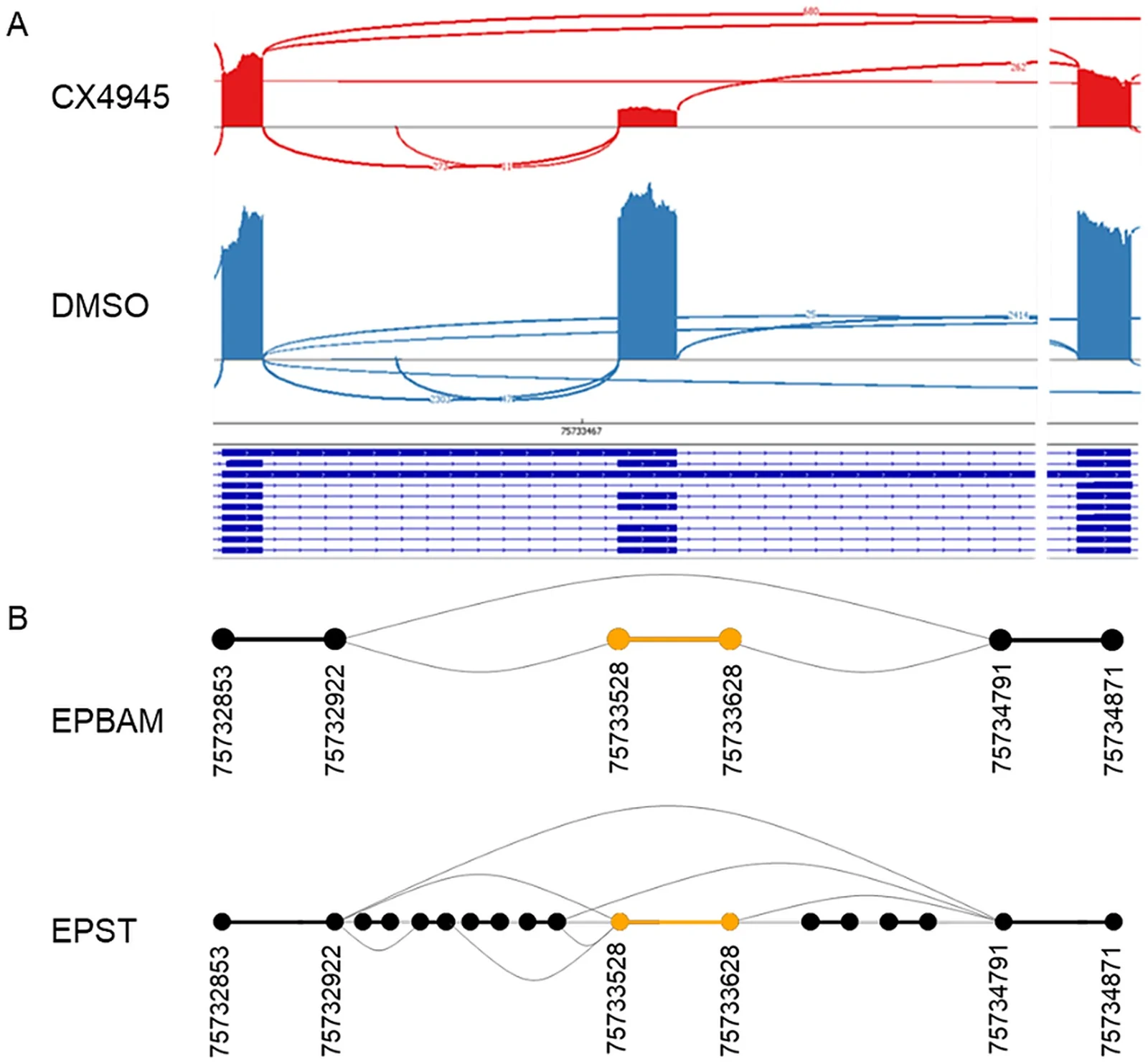
Sashimi plot, annotation, and SGs for the ACADM gene; the sashimi plot and the SGs were built as described in Fig. 6; to build the sashimi plot the BAM of all the samples for each condition (treated with CX4945 and untreated) were merged into a single one; the SG for EPBAM includes junction reads from the samples; on the contrary, the EPST SG is based on the annotation exclusively; in this case, the annotation is very complex and produces an intricate SG.

In other cases, such as CTC1, MRFAP1, PSMG1, RNF185, CPSF2, DONSON, KDM1B, and ZNF692, the complexity of the transcriptome prevents EPBAM, rMATS, or SUPPA2 from detecting some of these events, whereas EPST does detect them and correctly classifies them as ‘complex canonical’.

Finally, we highlight the ability of MAJIQ+VOILA to detect events: it detects 22 of the 23 events, classifying all of them correctly except for KIF20B. MAJIQ’s strategy of modelling all junctions allows it to identify events even in very complex splicing structures, conferring a clear advantage in this respect over the other methods. This performance is consistent with our SIRV analysis, where MAJIQ was also the tool that reported the largest number of events. However, unlike in the SIRV experiment—where a comprehensive ground truth allows us to estimate its FDR and reveals a substantial number of false positives—for the PCR dataset only 23 events were experimentally validated; consequently, it was not possible to determine the FDR for each of the methods.

Because each cell line was analysed as a separate five-versus-five contrast, the validated events were assessed three times independently. EPBAM detects 17 of the 23 events (20 event identifiers, since three genes contribute two events each); every one of them reaches *q* < 0.05 in at least two of the three cell lines and 19 of 20 in all three, with a concordant direction of ΔΨ in 95% of cases. Reproducibility across cell lines is therefore an observation rather than an assumption of the model.

We acknowledge three limitations of this validation. First, the events were pre-selected to be simple and amenable to PCR validation and may not represent the full complexity of the splicing landscape. Second, independent experimental validation of events uniquely identified by EPBAM or MAJIQ was not performed; such validation would strengthen confidence in *de novo* detection capabilities. Third, no negative controls—events chosen a priori as non-changing—were included in the original study, so a FDR cannot be estimated from this dataset for any tool, and we do not report one. This dataset should therefore be read as qualitative corroboration of the detection and classification comparison rather than as a quantitative benchmark; the false discovery estimates in this work rest on the SIRV, where the ground truth is complete, and on the simulated data.

## Discussion

This study presents a systematic evaluation of seven AS detection tools—rMATS, SUPPA2, MAJIQ+VOILA, EPST, Shiba, LeafCutter, and the newly introduced EPBAM—across simulated, SIRV under three annotation conditions (complete, incomplete, and overloaded), and RT-PCR-validated datasets. Within this benchmarking framework, we introduce EPBAM, which extends the EventPointer framework by constructing splice graphs from splice-aware alignments, enabling *de novo* event detection while retaining bootstrap-based statistical inference. EPBAM also introduces a least-squares optimization for effective-length estimation that accounts for uneven read coverage. Our evaluation reveals that no single tool dominates across all metrics, but that annotation quality, event definitions, and quantification strategies each have substantial and tool-specific effects on performance. We also verified that these conclusions are not specific to the thresholds chosen: sweeping the minimum |ΔΨ| and the significance cut-off on the SIRV leaves EPBAM among the methods with the lowest FDR in every annotation condition, and preserves its advantage under an IA (Supplementary Table S7, Supplementary Table S8, Supplementary Fig. S15, Supplementary Fig. S16, Supplementary Fig. S17; Supplementary Text S10).

### Event-locus detection

At the level of splicing loci rather than events—the fraction of the 87 true alternative SIRV junctions each tool interrogates—the same pattern holds: annotation-based tools drop from 96.6% (EPST) and 90.8% (SUPPA2) under CA to 52.9% and 51.7% under IA, whereas EPBAM stays at 88.5% and 83.9%. Under OA all tools recover: MAJIQ, EPBAM, and rMATS build less events on the junctions present only in the overloaded reference (10, 13, and 14, respectively, versus 20 for SUPPA2 and Shiba).

### The challenge of alternative splicing event definitions

The computational analysis of AS remains complex primarily due to the absence of a universally accepted definition of a splicing event. This lack of standardization complicates direct comparisons between computational pipelines, as each tool defines and categorizes AS events differently. While canonical events like exon skipping, alternative splice sites, and intron retention are widely recognized, the detection of non-canonical and complex events is inconsistent across methods. This variability underscores the necessity of understanding the specific event definitions and algorithms each tool employs when interpreting results, as these factors significantly influence the number and types of events detected.

rMATS, Shiba, and SUPPA2 primarily rely on predefined canonical splicing patterns, such as exon inclusion and exclusion patterns, which primarily encompass canonical events. While flexible, the absence of strict formal definitions can lead to classification variability. MAJIQ defines splicing through ‘Local Splicing Variations (LSVs)’, representing splits in a gene splice graph where multiple paths originate or converge. Its companion tool, VOILA, categorizes events based on these LSVs, allowing for the detection of both canonical and complex patterns. EventPointer (EPBAM and EPST) delineates splicing events by constructing a complete SG for each gene, defining each event with three distinct paths: alternative (PathA), constitutive (PathB), and a shared reference path (PathRef). This methodology enables the identification of complex events beyond canonical patterns and facilitates primer selection for validation.

These differences in event definitions create a fundamental challenge for cross-tool comparison. Our matching procedure (Supplementary Text S2) maps events from each tool but LeafCutter onto a path-pair framework using genomic coordinate overlap. While this enables systematic comparison, it cannot perfectly reconcile distinct event ontologies; consequently, the false-event counts of Table 3 are conditional on the matching framework rather than universal FDRs. We advocate for community efforts to establish standardized event ontologies and benchmarking frameworks that would enable more rigorous cross-tool evaluation.

### Impact of alternative splicing complexity on tool performance

The complexity of AS events significantly influences tool performance, with notable differences between simulated (low complexity) and experimental SIRV (moderate to high complexity) datasets.

SUPPA2 and rMATS, being primarily based on canonical patterns, perform well in low-complexity scenarios, such as simulated datasets, where gene annotation is complete. However, their event detection is more limited in high-complexity conditions (SIRV data), leading to a reduced ability to detect events and lower accuracy in estimating Ψ and ΔΨ. For instance, using OA, which increases AS complexity, decreased Ψ and ΔΨ correlation for SUPPA2, and rMATS showed a significant increase in FE detection.

MAJIQ and LeafCutter exhibited the lowest event detection rate with no false positives in low-complexity AS (simulated data). However, under high-complexity SIRV conditions, they showed an opposite trend, detecting a high number of events. Further, MAJIQ’s ΔΨ calculation approach demonstrated high correlation across all SIRV conditions, even improving under OA. This suggests that MAJIQ’s event definition is robust for ΔΨ quantification in complex contexts.

EPBAM and EPST performed well across both low- and high-complexity AS conditions. They nearly matched SUPPA2 and rMATS in simple splicing scenarios but showed more consistent performance across both low- and high-complexity conditions. Their performance also improved with increasing annotation complexity, especially for EPST, which showed an increase in detected events and improved Ψ and ΔΨ estimations under OA. Their flexible event definitions and ability to detect complex events make EPBAM and EPST highly robust for analysing AS across various complexity levels. Of course, the main advantage of EPBAM over EPST is its ability to discover novel events.

### The critical role of annotation quality

Annotation quality is a crucial factor in AS detection, influencing not only annotation-based tools but also *de novo* methods, which can exhibit biases due to their reliance on annotations during read alignment.

rMATS and MAJIQ utilize annotations to construct an initial SG, which is then expanded based on supported read junctions. In the case of Shiba, RNA-seq reads are first assembled into transcripts using a reference-guided assembler (StringTie2), and the resulting transcripts are merged with the reference annotation. While this preliminary SG construction improves computational efficiency by restricting processing to predefined regions, it may fail to detect some novel events. This preliminary reliance on annotations can lead to erroneous event identification if annotated isoforms are not expressed (e.g. in specific tissues or conditions). This was evident in the SIRV OA condition, where both Shiba and rMATS exhibited increased FE detection compared to the CA condition. Conversely, under IA, where more isoforms were expressed than annotated, their event detection capabilities were limited. This is particularly relevant in contexts like cancer, where aberrant AS may be induced, potentially hindering the detection of novel splicing events by predefined SGs.

EPBAM’s event detection is annotation-robust, since the annotation is used as a guide in the upstream alignment step by STAR (see Methods). However, the number of detected events varies only slightly across CA, IA, and OA (Table 3), and its performance is substantially more stable than that of other *de novo* tools, reflecting a reduced rather than eliminated dependency on annotation content. This approach, while computationally more demanding, demonstrated greater robustness across SIRV annotation conditions. The number of detected events matching SIRV reference annotations remained stable across CA, IA, and OA conditions, as did Ψ and ΔΨ estimations. This stability suggests that EPBAM’s method of SG construction is more resilient to annotation variability.

For tools that can leverage additional transcript information without overfitting—notably EPST and EPBAM—more comprehensive annotations can improve performance. However, this benefit is tool-dependent: SUPPA2’s Ψ correlation decreased under OA, and rMATS’s false positive rate increased substantially. We therefore do not make a universal recommendation for OAs, but emphasize that the optimal annotation strategy depends on the tool being used.

### Accuracy of Ψ and ΔΨ estimation

Ψ estimation shows a large dispersion across all methods; however, this error appears to be a systematic bias across analyses. Consequently, ΔΨ estimates are considerably more accurate, suggesting that comparative analyses between conditions (ΔΨ) provide more reliable insights despite biases in absolute Ψ quantification.

EPST consistently achieved the highest ΔΨ correlation among all tools, reaching up to *ρ* = 0.99 under OA conditions, and also attained the lowest RMSE values. Among *de novo* tools, EPBAM showed the largest ΔΨ correlation and lowest RMSE for all annotation conditions. MAJIQ also showed a significant increase in ΔΨ correlation compared to Ψ, comparable to EPBAM. LeafCutter Ψ and ΔΨ for introns also show a high correlation demonstrating a high degree of accuracy. An ablation of the coverage correction (Supplementary Text S11 and Supplementary Table S9) shows that estimating the coefficients improves the accuracy of both Ψ and ΔΨ, while the sensitivity analysis (Supplementary Text S9) shows that the results depend little on the value of λ.

### Differential event classification

We used the AUC to compare each tool regarding statistical classification. The AUC depends only on the ranking of events, it is comparable across tools regardless of their significance thresholds. EPBAM is the only one whose ability to recover truly changing events is unaffected by the annotation (0.827, 0.83, and 0.818 under CA, IA, and OA). In this analysis, for each tool, the events that matched SIRV were considered. Events with an increase in PSI of ˃0.2 in SIRV were classified as TP.

### Proposed hybrid alternative splicing analysis strategy

Considering the trade-offs between annotation-based and *de novo* approaches, an optimal AS analysis strategy could integrate both approaches.

Annotation-based tools (SUPPA2 and EPST) generally outperform their *de novo* counterparts in structured transcriptomes with low splicing complexity or perfect annotations, and are efficient for large datasets when the transcriptome is consistent across samples. *De novo* tools (EPBAM, rMATS, and MAJIQ) excel in detecting novel AS events and are more robust in high-complexity AS scenarios.

This hybrid strategy enhances transcriptome characterization, providing deeper insights into AS mechanisms and broadening research possibilities in transcriptomics by leveraging the strengths of both approaches. The analysis of real datasets (PCR validated) further illustrates the complementarity of each tool, showing that different tools may excel in classifying different types of events.

Based on our findings, we offer the following practical recommendations. First, when annotation quality is reliable and the focus is on well-characterized canonical events, annotation-based tools (EPST, SUPPA2) offer the highest quantification accuracy and computational efficiency. Second, when annotation quality is uncertain or novel event discovery is important—as in non-model organisms, disease contexts with aberrant splicing, or early-stage genome assemblies—*de novo* tools such as EPBAM provide greater robustness. Third, researchers should place greater confidence in ΔΨ estimates than in absolute Ψ values, as the former are more accurate across all tools. Fourth, combining annotation-based and *de novo* approaches can provide a more comprehensive view of the splicing landscape, leveraging the strengths of each.

Finally, the SIRV data proved to be a valuable benchmarking resource, providing externally validated ground truth for both Ψ and ΔΨ across events of varying complexity. Combined with the three-tier annotation framework (CA, IA, OA) introduced here, it enables a multi-dimensional evaluation of tool robustness that we recommend for adoption in future benchmarking studies.

EPBAM contributes to the landscape of AS tools by combining *de novo* event detection with annotation robustness and a principled coverage-corrected quantification framework. Its primary strengths—stable performance across annotation conditions and a low FDR for novel events—make it particularly suited for analyses where annotation quality is uncertain. Its foundation on design and contrast matrices, as in limma-based approaches to differential expression analysis [49], additionally supports experimental designs beyond simple two-group comparisons, a capability implemented in the EventPointer framework but not benchmarked in this study. However, our evaluation also shows that EPBAM does not uniformly outperform existing tools: annotation-based methods achieve higher absolute Ψ accuracy, and rMATS shows stronger concordance with PCR-validated canonical events. The choice of tool should therefore be guided by the specific analytical requirements of each study.

### Limitations

Several limitations of this study should be acknowledged. First, our simulation included only simple canonical events in two-isoform genes and did not evaluate performance across all event types or complex multi-isoform architectures. Second, we did not systematically vary sample sizes or biological variability, factors that may differentially affect tool performance. Third, while we included RT-PCR-validated data, independent validation of events identified by each of the tools was not performed. Fourth, LeafCutter’s intron-cluster representation does not map one-to-one onto the path-pair framework, and its calls are therefore interpreted at the level of intron clusters (Supplementary Text S1). VAST-TOOLS could not be included: it requires a precompiled, species-specific library of annotated events, which does not exist for the synthetic SIRV transcripts and cannot be generated through its standard pipeline, so the tool cannot be applied to our primary benchmark dataset. Fifth, the comparison with MAJIQ+VOILA is limited by fundamental differences in event definition and statistical framework: MAJIQ’s Bayesian posterior probabilities are not directly comparable to frequentist *P*-values, and its LSV-based events do not map one-to-one onto path-pair definitions. Sixth, EPBAM is computationally demanding relative to the other tools evaluated: constructing the full splice graph from aligned reads and bootstrapping at the read level required 27.8 h and 55 GB of peak memory for the 30-sample dataset analysed here, against 52 min and 5.7 GB for rMATS (Supplementary Text S8 and Supplementary Table S5). This cost is incurred once per dataset and is independent of the number of contrasts subsequently evaluated, but it is a practical constraint for large cohorts and a target for future optimization. We note also that we did not perform a computational scaling analysis; EPBAM’s construction of the full splice graph from aligned reads is more computationally demanding than annotation-constrained approaches, and future work should characterize its practical requirements.

In summary, this study provides a systematic evaluation of AS detection tools under varying annotation conditions, revealing that annotation quality, event definitions, and quantification strategies each have substantial and tool-specific effects on performance. The three-tier annotation framework (CA, IA, and OA) and the use of SIRV ground truth offer a multi-dimensional benchmarking approach that we recommend for future tool evaluations. Within this framework, EPBAM demonstrates competitive performance with particular robustness to annotation quality and reliable *de novo* event detection, complementing the strengths of existing tools.

**Key Points**

- We systematically benchmark EventPointer BAM (EPBAM) against widely used tools (EventPointer ST (EPST), replicate Multivariate Analysis of Transcript Splicing (rMATS), Super-fast Pipeline for alternative splicing analysis, version 2 (SUPPA2), Modeling Alternative Junction Inclusion Quantification + Visualization Of Inferred Local splicing Alternatives (MAJIQ+VOILA), and others), using both synthetic and experimental validation datasets, including Lexogen Spike-In RNA Variant (SIRV) and PCR-validated events.
- Lexogen SIRV, with known transcript composition and defined Ψ and ΔΨ values under complete, incomplete, and overloaded annotations, provide an externally validated ground truth to assess both accuracy and annotation robustness of alternative splicing (AS) tools.
- No single tool dominates across all evaluation metrics, highlighting the importance of dataset diversity and standardized benchmarking practices for meaningful tool comparison in the AS field.
- EPBAM is an event-based method for detection and quantification of AS that builds splice graphs from splice-aware alignments, enabling *de novo* event discovery while retaining bootstrap-based statistical inference.

## Supplementary Material

Supplementary_materials_bbag556

## Data Availability

The EventPointer package for R and all datasets, scripts, and supplementary materials necessary to replicate the results presented in this study are accessible at Figshare: http://dx.doi.org/10.6084/m9.figshare.28417043. A complete vignette of how to use EPBAM is available in Bioconductor at: https://www.bioconductor.org/packages/release/bioc/vignettes/EventPointer/inst/doc/EventPointer.html.
