# Supplementary material for: EPBAM: annotation-robust detection of alternative splicing events and a systematic benchmarking of current tools
Source: Brief Bioinform. 2026 Oct 8;27(5):bbag556. doi: 10.1093/bib/bbag556 (PMC13647278; doi:10.1093/bib/bbag556)
Supplement: Supplementary file 1 [file supplementary_materials_bbag556.zip › Supplementary_file_1_corrections_minor.docx]

**EPBAM: annotation-robust detection of alternative splicing events and a systematic benchmarking of current tools.**

**Supplementary Text, Tables and Figures**

**AUTHORS**

Cesar Lobato-Fernandez^1^, Laura Jareño^1^, Angel Rubio ^1^ and Juan A. Ferrer-Bonsoms^1,^*

^1^ Biomedical Engineering and Science Department, TECNUN, Universidad de Navarra, San Sebastián, Spain

* Correspondence and requests for materials should be addressed to J.A.F-B. (email: jafhernandez@unav.es)

**Supplementary Text 1: EVENT DEFINITION**

**Canonical events.** A cassette exon involves the selective inclusion or exclusion of an exon (Figure S1, A) within the mRNA. Intron retention (Figure S1, B) refers to the retention of an intron that is usually spliced out, leading to its inclusion in the mature mRNA. Alternative 5' and 3' splice sites modify the boundaries of exons (Figure S1, C-D), resulting in different transcript variants depending on which splice site is utilized. Alternative first and last exons arise from the use of different promoters or polyadenylation sites, influencing the transcript's start and end points, respectively (Figure S1, E-F). Mutually exclusive exons describe a scenario where only one of two possible exons are included in the final mRNA transcript, ensuring that the exons are never simultaneously present (Figure S1, G).

**Figure S1:** Graphical representation of the 7 types of canonical splicing events. Constitutive exons are depicted as blue boxes, while alternative sequences are shown as striped boxes. (A) Cassette exon: an exon that may be either included or excluded from the mRNA. (B) Retained intron: an intron remains within the mRNA. (C, D) Alternative 5’ splice site and alternative 3’ splice site: different splice sites at the 5’ or 3’ end of an exon is used, respectively. (E, F) Alternative first exon and alternative last exon: these involve the selection of different promoters and alternative polyadenylation sites, respectively. (G) Mutually exclusive exons: only one of two consecutive exons are included in the mRNA, not both.

Canonical splicing events are characterized by the presence of two distinct paths: the constitutive path, which follows the standard splice site usage, and the alternative path, which involves the selective alteration of these sites to produce diverse mRNA isoforms.

Complex splicing events exhibit more intricate and variable patterns, often involving multiple exons and introns. These events generate splicing isoforms with complex structures that are challenging to standardize, making them less readily detectable by conventional AS analysis tools (1). For instance, a double cassette exon can be classified as a complex event, as can a cassette exon that simultaneously exhibits alternative 5' or 3' splice sites. Additionally, some complex events involve even more intricate exon-intron interactions, further complicating their classification and detection.

**Splicing events for each tool.** Within the general framework of what constitutes an AS event, each tool must establish its own specific definition to implement a computational methodology capable of capturing AS dynamics. The SG plays a central role in defining and detecting AS events. Each tool employs a unique approach to constructing the SG, tailored to align with its computational interpretation of AS events. From this SG, each tool determines the paths for the events—PathA (alternative path) and PathB (constitutive path)—, as described in the Canonical AS Events Definition section above (Figure S1). The only exception is LeafCutter, whose unit of analysis is an intron cluster rather than a path pair; it is described separately at the end of this section.

rMATS, SUPPA2, and Shiba do not provide formal definitions of splicing event; however, all three utilize the concepts of exon inclusion and exclusion to define their event detection patterns (2, 3). This approach facilitates a flexible and broad detection of splicing events, encompassing the canonical types (See Table 1). The three tools differ mainly in how the underlying SG that generates these inclusion/exclusion paths is built, and consequently in whether an event can be detected without prior annotation support. SUPPA2 initiates event detection using the GTF annotation alone, so its candidate paths are limited to those implied by annotated isoforms, whereas rMATS also starts from the GTF but includes an additional step that employs junction reads to detect de novo events that conform to its canonical event patterns even when the alternative path is absent from the annotation (Figure S2, B–C). These patterns are adaptable, allowing for event categorization even when annotation variability is present. Shiba (16) incorporates unannotated evidence a priori rather than as a separate post-hoc step: RNA-seq reads are first assembled into transcripts using a reference-guided assembler (StringTie2), and the resulting transcripts are merged with the reference annotation before the SG is derived. Despite this flexibility, the absence of strict formal definitions can lead to variability in event classification, contingent on the specific criteria and thresholds set by the user in any of the three tools.

EventPointer delineates splicing events by constructing a complete SG for each gene. Unlike rMATS and SUPPA, EventPointer defines that each event is composed of three distinct paths: PathA, PathB and a third path known as the reference path (4, 5). This reference path includes the regions shared by the isoforms that include either PathA or PathB within the SG (Figure S2, D). This methodology allows for the identification of complex events that extend beyond canonical splicing patterns, providing a more comprehensive representation of splicing diversity. On the other hand, the presence of a reference path makes it easier to automatically select primers to validate the events.

MAJIQ studies splicing through the concept of local splicing variations (LSVs). According to its developers, “LSVs can be defined as splits in a gene splice graph converging into or diverging from a reference exon” (6). In the splice graph, an LSV corresponds to a node from which multiple splice paths originate (single source LSV, or SS-LSV) or into which multiple paths converge (single target LSV, or ST-LSV) (7) (Figure S2, E). The edges in MAJIQ’s splice graph represent possible splicing junctions that emerge from or lead to each LSV node, capturing the diversity of splicing choices. MAJIQ’s companion tool, VOILA, utilizes this splice graph to categorize splicing events and assign specific AS types based on the junctions and LSVs identified. The flexible structure of MAJIQ’s splice graph allows VOILA to identify both canonical AS events and more complex splicing patterns, offering an adaptable framework for detecting intricate splicing architectures.

**Figure S2** : The figure shows the structure of a gene with three different transcripts. Two of them are known and the third is novel. It also includes a (A) representation of aligned RNA-Seq reads mapped to gene annotations. Exons are shown as blue boxes, with aligned reads represented by color-coded segments. (B) SG construction where nodes (black circles) represent exons (e) and edges (j) denote junctions. Local splicing variations (LSVs) are identified, including single source LSV (LSVSS) and single target LSV (LSVST), with a potential de novo junction (j4) indicated. (C) Event detection patterns for SUPPA2, rMATS, and Shiba illustrating their classification of AS events using Paths (Path1, Path2, Path3). SUPPA2 identifies possible cassette exons, while rMATS and Shiba detect specific canonical cassette exon events. (D) EventPointer’s splicing event definition, based on three paths: PathA (alternative), PathB (constitutive), and PathRef (reference). This methodology supports the detection of both complex and canonical events. (E) MAJIQ’s LSV-based event detection. The splice graph highlights LSVSS and LSVST nodes and junctions (j1–j4), with VOILA assigning AS event types, including complex events.

LeafCutter (17) constructs an SG as well, but with a fundamentally different convention from the other tools discussed here. Rather than representing splice-site genomic positions as nodes, LeafCutter's SG is built directly from split reads: each node is an intron inferred from a spliced alignment, and an edge connects two introns whenever they share a donor or acceptor splice site, so that edges represent the junction reads supporting each intron rather than exons. The connected components of this intron-based graph define intron clusters, and, following an iterative filtering step that discards rarely used introns, each cluster becomes LeafCutter's unit of analysis. Consequently, LeafCutter does not label individual events as canonical types such as SE, A5SS, A3SS, RI, or MXE; a single cluster can instead capture a splicing choice of arbitrary complexity, and, because clusters are built exclusively from observed junction reads, the same definition applies without distinction to annotated and novel excision patterns. A further consequence of relying exclusively on split reads is that LeafCutter does not report intron retention, since a retained intron produces no spliced alignment.

**Figure S3:** LeafCutter's intron-based definition, illustrated on the same toy gene and read alignments as Figure S2. (A) Aligned RNA-Seq reads mapped to gene annotations and the corresponding GTF gene annotation, reproduced from Figure S2A; Transcript isoform 1 and Transcript isoform 2 are annotated, while the third transcript is novel (hatched boxes). In this case, only junction reads are shown, as LeafCutter relies exclusively on these reads. (B) LeafCutter's splicing-graph convention applied to the junction reads (j1–j4). Each junction read defines an intron: I1 and I2 are the two junctions of the full-inclusion isoform, I3 is the annotated skipping junction supported by Transcript isoform 2, and I4 is the de novo skipping junction supported only by reads (the novel transcript in A). Unlike the exon/splice-site graph used by the other tools (Figure S2B), an intron here is not an edge connecting nodes; each intron instead becomes a node once collapsed into the graph shown in (C). (C) Introns sharing a donor splice site (I1 and I4, at 2.b) or an acceptor splice site (I2, I3 and I4, at 4.a) are connected by an edge; the resulting connected component, comprising I1–I4, defines an intron cluster — LeafCutter's unit of analysis, quantified as the excision proportion of each intron relative to the total read count of the cluster.

Differences in the methodologies used to define splicing events complicate the comparison of coincident events between AS detection tools. Variations in event definitions and reference positions can lead to discrepancies in event categorization and alignment (Figure S2). These inconsistencies are further influenced by how tools interpret junction reads or GTF-based annotations. Figure S2 illustrates a simple case of the difficulties in matching the events provided by different tools. However, any strict canonical events coincide in its labelling by all the tools. The detailed methodology for matching splicing events between tools and the experimental datasets is provided in the Supplementary Text 2: "Matching Events".

**Supplementary Text 2: Matching events**

**Matching of Splicing Events with SIRV**

For every tool, each event was rewritten as a pair of alternative paths, Path A and Path B, together with the reference path shared by both (Figure S2). This representation is immediate for EPST and EPBAM, which define events in these terms, and almost immediate for rMATS, SUPPA2 and Shiba, which report the two alternatives as resolved genomic coordinates. For MAJIQ+VOILA it is not immediate in all cases: most of the categories returned by modulize map onto a pair of paths, but multi-exon spanning and alternative intron events describe the event through its junctions rather than through the exons of each alternative, and were matched by exact coordinate equality against the junctions of the SIRV transcripts (Figure S5). LeafCutter is the only tool for which this representation cannot be derived from its output, and is treated separately below.

The ranges corresponding to Path A and Path B were delineated and matched against SIRV transcripts (Figure S4, B). All sequence ranges annotated as exons in the GTF were considered as reference SIRV regions for this matching process (Figure S4, A).

Once the ranges for Paths A and B were established for each event, a matching process between these ranges and the SIRV-associated regions was performed using the findSpliceOverlaps function from the R package GenomicAlignments (8) (Figure S4, C).

If both paths of an event were compatible with at least one SIRV transcript, the event was considered a “match” with SIRV, indicating compatibility with splicing events present in SIRV transcripts. Otherwise the event was counted as a false event, meaning that its structure does not correspond to any splicing choice among the isoforms present in the mixes. Further, if any of the paths matched transcripts were not included in the reference SIRV region, the event was classified also as de novo (Figure S4, D).

LeafCutter does not define events and was matched at the intron level. Within each cluster containing at least two introns, the transcripts supporting a given intron are those that contain it exactly, and the alternative transcripts are those using any other intron of the same cluster; introns for which either group was empty were discarded.

**Matching Events Between Tools**

The criteria for considering two events from different tools as equivalent are as follows:

- The genomic positions of PathA and PathB from the respective events identified by different tools must overlap. This is determined using the findOverlaps function from the GenomicAlignments R package. Since the A and B paths naming is somewhat arbitrary, it is also checked if path A from a tool matches with the B path from a different tool and vice versa.
- The overlapping paths, as defined by the previous criterion, must be compatible with the splicing patterns of the annotated SIRV transcripts.

According to the matching criteria employed, only those events that are initially validated through matching with SIRV can be considered for cross-tool event matching.

**Matching Predicted Splicing Events with PCR-Validated Events**

To evaluate the concordance between computational predictions and experimental validation, we implemented the following criteria to match splicing events detected by each tool with those assessed by endpoint PCR:

- From the original set of 29 PCR-validated events, 6 were excluded due to insufficient validation consistency or noisy banding patterns that could compromise event identification and quantification.
- Genomic coordinates corresponding to PCR amplicons were converted to the reference genome version used in this study using UCSC liftOver.
- A computational event was considered a potential match if its genomic region overlapped with the region targeted by the PCR assay.

**Matching Events with Simulated Data**

The ground truth of the simulated dataset is a set of splicing events defined by SUPPA event identifiers, split beforehand into positives (|ΔΨ| > 0.2) and negatives (|ΔΨ| ≤ 0.2), and into cassette-exon and alternative 5′/3′ splice-site events. Because a SUPPA identifier encodes the chromosome and the coordinates of the two alternative junctions, each ground-truth event was converted into the same Path A / Path B representation used throughout this work: Path A comprises the two flanking splice-site anchors together with the body of the alternative exon, and Path B the two flanking anchors alone.

For every tool except LeafCutter, the events reported were brought into the SUPPA's terms. For LeafCutter, the ground truth was expressed in LeafCutter's terms: each event was decomposed into its constituent junctions — the two alternative junctions in alternative 5′/3′ events, and those two plus the implicit skipping junction in cassette-exon events, which together are exactly the intron cluster that LeafCutter builds for a cassette exon. An event was considered detected when a single LeafCutter cluster contained those junctions.

**Figure S4:** A) SIRV transcript exonic ranges from the GTF annotation file. SIRV107 is not included in the incomplete annotation GTF file. B) Cassette exon (red) and Alternative 5’ (blue) events detected in SIRV samples. C) Matching between SIRV exonic annotation ranges and ranges derived from the events in (B), using the findSpliceOverlaps function. D) Top: Resulting match table from the function, highlighting de novo event detection in yellow. Bottom: PSI calculation for SIRV events based on transcript matches with each splicing event.

**Figure S5:** Canonical splicing events and complex splicing events identified by MAJIQ+VOILA. Left column: Canonical splicing events including alternative 3' splicing, retained introns, alternative first and last exons, and mutually exclusive exons, with corresponding PathA and PathB ranges. Right column: Complex splicing events detected by MAJIQ+VOILA, including tandem cassette exons, alternative 5'/3' events, putative alternative 5'/3' events, and multi-exon spanning events, with detailed PathA and PathB ranges.

**SIRV isoforms per gene**

**Table S1:** Number of isoforms per gene in SIRV annotations.

|  | **Nº of isoforms per gene** | | |
| --- | --- | --- | --- |
|  | Complete annotation (CA) | Incomplete annotation (IA) | Overload annotation (OA) |
| **SIRV1** | 8 | 5 | 12 |
| **SIRV2** | 6 | 3 | 9 |
| **SIRV3** | 11 | 8 | 15 |
| **SIRV4** | 7 | 5 | 11 |
| **SIRV5** | 12 | 7 | 17 |
| **SIRV6** | 18 | 12 | 25 |
| **SIRV7** | 7 | 4 | 11 |
| **Total** | 69 | 44 | 100 |

**Table S2:** Number of junctions reported in each annotation. Junctions shared by all transcripts are considered constitutive and alternative otherwise. Missing indicates the splice junctions absent from the IA and Extra the additional junctions present in the OA.

|  | **Junctions** | **Constitutive** | **Alternative** | **Missing** | **Extra** |
| --- | --- | --- | --- | --- | --- |
| **CA** | 114 | 27 | 87 | 0 | 0 |
| **IA** | 89 | 43 | 46 | 41 | 0 |
| **OA** | 138 | 27 | 111 | 0 | 24 |

**Figure S6:** Venn diagram and upset plot of SIRV overload annotation matching events from different tools.

**Figure S7:** Venn diagram and upset plot of SIRV incomplete annotation matching events from different tools.

**Figure S8:** ROC curves for each tool in SIRV.

**Supplementary Text 3:** $\boldsymbol{\Psi}$ **Calculation**

The Percent Spliced In ($\Psi$) metric is the standard measure for quantifying AS events. Its calculation varies across tools, reflecting differences in the definition of AS events and alignment strategies.

For tools like SUPPA2 and EPST, the $\Psi$ value is derived from isoform abundances based on genome annotations. Let N represent the number of isoforms that include PathA (alternative) and M the number of isoforms for PathB (constitutive). The abundance of the n-th isoform in PathA is denoted as $\left[ T_{A}^{n} \right]$ and the abundance of the m-th isoform in PathB as$\left[ T_{B}^{m} \right]$. The $\Psi$ value is then calculated as follows:

$\Psi_{SUPPA/EPST}=\frac{\sum_{n=1}^{N} \left[ T_{A}^{n} \right]}{\sum_{n=1}^{N} \left[ T_{A}^{n} \right]+\sum_{m=1}^{M} \left[ T_{B}^{m} \right]}$,	(1)

In Equation (1), the denominator for EPST is, by definition, identical to the sum of the isoforms that include the reference path.

For tools that rely on splice-aware aligners, such as rMATS and EPBAM, the $\Psi$ value is calculated using read counts obtained from the alignment process. The expression for Ψ in these tools can be simplified as follows:

$\Psi_{rMATS/EPBAM}=\frac{{R_{A}/l}_{A}^{*}}{{R_{B}/l}_{B}^{*}+{R_{A}/l}_{A}^{*}}$,	(2)

where $R_{A}$ and $R_{B}$ are the read counts associated with each path, and $l_{A}^{*}$ and $l_{B}^{*}$ are the effective lengths associated with each path.

The main difference between rMATS and EPBAM tools lies in the calculation of effective lengths. In rMATS, these effective lengths depend on, the length of each path, the read length, the fragment length, and the type of event detected as each type of event has associated path patterns (Supplementary from (2)). For EPBAM, the effective length is computed by solving a penalised least squares problem also considering the reads in the reference path. EPBAM’s approach exploits the redundancy of the information provided by the reference path. The sum concentration of the isoforms in the reference path must be identical to the concentration of the isoforms in paths A and B. This restriction can be softly included in the Ψ estimation by modifying the equivalent lengths (see section “ $\Psi$ Calculation for EPBAM” of Supplementary Text 3).

Shiba follows the same general form as Equation (2) but replaces the effective-length-normalised abundance with raw exon-exon and exon-intron junction read counts pooled across the replicates of each group. Because every junction read spans a splice site regardless of the length of the flanking exon or intron, all junction reads have the same effective length ($l_{A}^{*}$ = $l_{B}^{*}$ = constant), so this term cancels out of Equation (2), which reduces to a direct ratio of junction read counts. For a skipped-exon or retained-intron event, A is the mean of the upstream and downstream inclusion-junction read counts and B is the exclusion-junction read count.

LeafCutter departs from the two-path formulation used above: rather than a fixed PathA/PathB pair, its unit of analysis is a cluster of two or more alternatively excised introns sharing a donor or acceptor splice site. For each intron k within a cluster of K introns, Ψ is defined as the proportion of the cluster's total junction read count supported by that intron:

$\Psi_{\mathrm{LeafCutter}\left( \mathrm{intron}_{k} \right)}=\frac{R_{k}}{\sum_{j=1}^{K} R_{j}}$	(3)

where $R_{j}$ is the number of split reads supporting intron j. When K = 2, Equation (3) reduces to the same A/(A+B) form as Equation (2); for K > 2 it instead yields one Ψ value per intron rather than a single Ψ for the cluster. As in Shiba, $R_{j}$ are raw junction read counts: because every junction read spans a splice site independently of intron length, no effective-length correction is required.

MAJIQ quantifies LSVs by estimating the relative inclusion level (RIL) of each junction within the LSV. In canonical events —where LSVs are involved in only a single event— this quantification is equivalent to calculating the exon’s percent spliced in (PSI, or $\Psi$). For more complex LSVs involving three or more potential splice paths, MAJIQ calculates the marginal inclusion level (MIL) for each individual junction. It is important to note that MAJIQ only uses the junction reads to compute the relative inclusion levels. We have summarized the $\Psi$ value for each module by taking the average of the MIL values of the junctions in the module that belong to the same path. In section (“$\Psi$ Summarization for MAJIQ” of Supplementary Text 3) we show the formulas and a workout example on how the summarization is done.

$\boldsymbol{\Psi}$ **Calculation for SIRV**

Since SIRV is extensively used for validation in the main manuscript, we will describe their definition of splicing events. SIRV uses a hypothetical reference transcript that includes all the transcribed regions of a gene. Each actual transcript is compared against this reference. Using the definitions of canonical splicing events, each difference between the transcript under study and the reference is labeled as a specific alternative splicing event. Since the reference includes all possible transcribed regions, there are no "intron retention" events—they are retained in the reference—. SIRV introduces a new type of event, known as exon splitting events— which is the counterpart of an exon retention event —.

Given the known abundance ratios of the transcripts in each SIRV mix —A(SIRV) —, the Ψ calculation for an event $"e"$ from a ${Tool}_{i}$ where $i\in\left\{ EPBAM,EPST,MAJIQ,SUPPA2,rMATS \right\}$ is performed as following:

$\Psi_{SIRV|{Tool}_{i}}^{e}=\frac{\sum{A(SIRV)\in PathA}_{{Tool}_{i}}^{e}}{\sum A\left( SIRV \right)\in\left\{ {PathA}_{{Tool}_{i}}^{e}, {PathB}_{{Tool}_{i}}^{e} \right\}}$	(4)

Each AS software detects different splicing paths for a given event; consequently, even when two software tools identify the same event “e”, their designated paths “A” and “B” may differ. As a result, the theoretical Ψ values calculated for SIRV events depend on the specific transcripts assigned to pathways “A” and “B” by each software ($\Psi_{SIRV|EPBAM}^{e}\neq\Psi_{SIRV|rMATS}^{e}$).

$\boldsymbol{\Psi}$ **Summarization for MAJIQ+VOILA**

MAJIQ's LSV quantification is based on estimating the relative inclusion level of each junction in the LSV. For a given junction $ji$ within the set of junctions $\{j_{1},j_{2},\ldots,j_{L}\},$ the inclusion proportion $\Psi_{ji}$ is defined as:

$\Psi_{ji}=\frac{R_{ji}}{\sum_{l=1}^{L} \left[ R_{jl} \right]}$	(5)

where $L$ is the total number of junctions in the LSV, and $R_{ji}$ represents the read count abundance associated to the junction $j_{i}$.

To calculate the overall splicing inclusion level $\Psi$ for an event, we first compute the average $\Psi$ for each path associated with the event. For path A, representing the alternative path linked to the event, the mean inclusion level is calculated as:

$\bar{\Psi}_{A}^{MIL}=\frac{\sum_{i=1}^{I} \left[ \Psi_{ji} \right]}{I}$	(6)

where $\Psi_{ji}\in{\{\Psi}_{j1},\Psi_{j2},... \Psi_{jI}\}$ corresponds to the$\Psi$ values of each junction in path A, and $I$ is the total number of junctions that constitute path A. Similarly, the same computation applies to path B, considered the constitutive path. The total $\Psi$ for the splicing event is then estimated as:

$\Psi_{MAJIQ}^{event}=\frac{\bar{\Psi}_{A}^{MIL}}{\bar{\Psi}_{A}^{MIL}+\bar{\Psi}_{B}^{MIL}}$	(7)

Let us include a workout example of how these values are computed. In MAJIQ-VOILA, each splicing event is composed of two Local Splicing Variants (LSVs): A Start-LSV (LSVSS) and a Target-LSV (LSVST). The LSVSS represents alternative donor sites connecting to different acceptors, while the LSVST represents alternative acceptor sites receiving connections from different donors. These LSVs define the alternative splicing landscape by capturing the different ways exons can be connected. Let’s assume the gene shown in Figure S9 with a Cassette Exon event:

**Figure S9:** Proposed summarization of PSI in MAJIQ-VOILA.

The number that appears in each junction is the number of junction-reads mapped to it. MAJIQ analyzes splicing by considering each junction in two contexts: the LSVSS and the LSVST. In the LSVSS we have the following values for $\Psi$, which are computed according to Equation 4:

$$\Psi_{j1}=\frac{R_{j1}}{R_{j1}+R_{j2}+R_{j3}}=\frac{20}{20+15+5}=0.5$$

$$\Psi_{j2}=\frac{15}{20+15+5}=0.375$$

$$\Psi_{j3}=\frac{5}{20+15+5}=0.125$$

The two junctions in the LSVST will have the following values:

$$\Psi_{j4}=\frac{20}{20+18}=0.53$$

$$\Psi_{j3}=\frac{18}{20+18}=0.47$$

Notice that junctions 1 and 4 are the same junction, however, MAJIQ consider different Ψ depending on the LSV under study. PathA includes j1-j4. The corresponding summarized $\bar{\Psi}_{A}^{MIL}$ is:

$$\bar{\Psi}_{A}^{MIL}=\frac{\sum_{i=1}^{I} \left[ \Psi_{ji} \right]}{I}=\frac{0.5+0.53}{2}=0.515$$

PathB includes junctions j2 and j5:

$$\bar{\Psi}_{B}^{MIL}=\frac{0.375+0.47}{2}=0.4225$$

Our proposed summarized $\Psi_{MAJIQ}^{event}$ value according to Equation 4 is:

$$\Psi_{MAJIQ}^{event}=\frac{\bar{\Psi}_{A}^{MIL}}{\bar{\Psi}_{A}^{MIL}+\bar{\Psi}_{B}^{MIL}}=\frac{0.515}{0.515+4225}=0.55$$

$\boldsymbol{\Psi}$ **Calculation for EPBAM**

$\Psi$ metric in EPBAM is calculated using the contributions of three distinct paths: ${Path}_{A}$ (alternative), ${Path}_{B}$ (constitutive), and ${Path}_{Ref}$ (reference). The calculation employs a non-negative least squares (NNLS) optimization algorithm (9) to estimate the relative contributions of ${Path}_{A}$ and ${Path}_{B}$, adjusted by their effective lengths and a regularization parameter,$\mu$.

Since the efficiency of the sequencing for different loci of the transcriptome is not uniform, it can be naïve to assume that the concentration of the isoforms for each path is simply proportional to the number of reads of the path and its equivalent length. EPBAM includes a reference region shared by all the isoforms that include either the ${Path}_{A}$ or the ${Path}_{B}$. This reference region enforces a soft restriction that helps estimate each path's concentration.

For each sample i out of a total of n samples:

$\left[ {{TRef}_{est}}_{i} \right]=\left[ {{TA}_{est}}_{i} \right]+\left[ {{TB}_{est}}_{i} \right]$,	(8)

Where ${{TRef}_{est}}_{i}$ is the sum of the concentrations of the isoforms in the reference region for sample i, and ${{TA}_{est}}_{i}$ and ${{TB}_{est}}_{i}$ are the sum of the concentrations of the isoforms in the path A and B respectively for sample i. These concentrations are unknown in EPBAM. It can be assumed that the concentration of the isoforms is proportional to the number of reads ($R_{A},R_{B},R_{Ref}$) of each path (${Path}_{A}$, ${Path}_{B}, {Path}_{Ref}$), albeit the constant of proportionality needs not to be the equivalent length:

${{TRef}_{est}}_{i}\propto R_{{Ref}_{i}}, {{TA}_{est}}_{i}\propto R_{A_{i}}, {{TB}_{est}}_{i}\propto R_{B_{i}}$	(9)

In an ideal case, the constants of proportionality for each of them would be the effective lengths of ${Path}_{A}$, ${Path}_{B}$, and ${Path}_{Ref}$: $1/l_{A}$, ${1/l}_{B}$ and $1/l_{Ref}$.

Using this information, it is possible to solve:

$R_{Ref}\cdot p= R_{A}\cdot w +R_{B}\cdot y \to R_{Ref}= R_{A}\cdot\frac{w}{p} +R_{B}\cdot\frac{y}{p}$	(10)

The scaling coefficients for ${Path}_{A}$ and ${Path}_{B}$ are $u=\frac{w}{p}$ and $v=\frac{y}{p}$ , respectively. Its expected value in an ideal case is ${l_{Ref}}/{l_{A}}$ and ${l_{Ref}}/{l_{B}}$ respectively.

In this case, $R_{Ref}$, $R_{A}$ and $R_{B}$ are known and $u$ and $v$ are the unknowns. It is possible to solve for $u$ and $v$ using a least squares approach:

$\min_{x\geq0} \left\| Ax-b \right\|$	(11)

Where:

- $A=\left[ \begin{matrix} R_{A} & R_{B} \end{matrix} \right]$ is a matrix ($A\in\mathbb{R}^{n\times2}$) where n is the number of samples.
- $b=R_{Ref}$ is a ($b\in\mathbb{R}^{n\times1}$).
- $x=\left[ \begin{matrix} u \\ v \end{matrix} \right]$ is a ($x\in\mathbb{R}^{2\times1}$) vector represents the scaling coefficients for ${Path}_{A}$ and ${Path}_{B}$, respectively.

$\boldsymbol{\Psi}$ **Calculation Without Regularization** $\mathbf{(}\boldsymbol{\mu}\mathbf{=0)}$

If $\mu=0$, the NNLS (non-negative least squares) algorithm directly solves:

${\min_{x\geq0} \left\| Ax-b \right\|}^{2}$	(12)

The resulting coefficients $u$ and $v$ are used to estimate the contributions from ${Path}_{A}$ and ${Path}_{B}$: ${TA}_{est}=R_{B}u$, ${TB}_{est}=R_{B}v$ .

Finally, the estimated value for $\Psi$ is

$\Psi_{EPBAM}= \frac{{TA}_{est}}{{TA}_{est} + {TB}_{est}}$	(13)

$\boldsymbol{\Psi}$ **Calculation with Regularization** $\mathbf{(}\boldsymbol{\mu}\boldsymbol{\neq0)}$

It could be advisable, for example for experiments with very few samples, to introduce a prior on the values of $u$ and $v$. We have included a penalized NNLS problem to account for this possibility. A penalty term $\mu$ is introduced to ensure that the estimated contributions of ${Path}_{A}$ and ${Path}_{B}$ align with their expected proportions relative to ${Path}_{Ref}$. The adjusted system augments 𝐴 and 𝑏 to include the penalty terms. The complete matrix system becomes:

$\tilde{A}=\left[ \begin{matrix} R_{A} & R_{B} \\ \mu& 0 \\ 0 & \mu\end{matrix} \right], \tilde{b}=\left[ \begin{matrix} R_{Ref} \\ \mu\cdot\frac{l_{Ref}}{l_{A}} \\ \mu\cdot\frac{l_{Ref}}{l_{B}} \end{matrix} \right]$	(14)

The augmented system solves:

${\min_{x\geq0} \left\| \tilde{A}x-\tilde{b} \right\|}^{2}$	(15)

If $\mu=0$, this system is equivalent to the previous case. If $\mu$ is extremely large, $u$ and $v$ will be equal to the priors, i.e. $\frac{l_{Ref}}{l_{A}}$ and $\frac{l_{Ref}}{l_{B}}$ respectively. We selected $\mu$ to be equal to:

$\mu=\sqrt{\lambda\cdot\frac{\left\| b-Ax \right\|_{2}^{2}}{\varepsilon+\sum\left( x-\left[ \begin{matrix} \frac{l_{Ref}}{l_{A}} \\ \frac{l_{Ref}}{l_{B}} \end{matrix} \right] \right)^{2}}}$	(16)

Where the default value of $\lambda$ is 0.1 and $x$ is the solution of the unregularized system.

**Final** $\boldsymbol{\Psi}$ **Calculation**

Using the estimated contributions from the adjusted system:

${TA}_{est}=R_{B}u{, TB}_{est}=R_{B}v$	(17)

The $\Psi$ is computed as:

$\Psi_{EPBAM}= \frac{{TA}_{est}}{{TA}_{est} + {TB}_{est}}$	(18)

This method ensures that the $\Psi$ calculation integrates the shared contributions of ${Path}_{Ref}$, while the regularization term ($\mu$) improves robustness by incorporating the relative effective lengths of the paths. By leveraging NNLS, EPBAM achieves a precise and balanced quantification of splicing inclusion levels, particularly for complex events where ${Path}_{Ref}$ plays a critical role. Supplementary Text 9 analyses the sensitivity to λ within its working range, and Supplementary Text 11 its limiting values: the default λ = 0.1 against λ → ∞, where Equation (18) reduces to Equation (2).

**Supplementary Text 4: P-value Calculation**

Statistical Analysis

Once the $\Psi$ value is estimated, most software tools (including all the ones selected for the comparison) provide a statistical analysis to state the significance of the changes across the different conditions. rMATS, SUPPA2, Shiba and EP provides a single value of $\Psi$ for each event. On the contrary, MAJIQ provides two or more values of RILs for each LSV, that in turn are summarized in this work using the procedure described in the supplementary material. LeafCutter, in turn, reports a single p-value per cluster of two or more alternatively excised introns, together with an individual ΔΨ for each intron in the cluster.

In rMATS, hypothesis testing evaluates whether the difference in Percent Spliced In ($\Psi$) values ($\Delta\Psi$) between two sample groups exceeds a user-defined threshold, *c*, against the null hypothesis that no such difference exists. This approach facilitates the comparison of alternative splicing events between two distinct conditions by integrating both statistical significance and biological relevance.

Incorporating a $\Delta\Psi$ threshold effectively filters out minor splicing changes that, while statistically significant, may lack biological importance. In contrast, traditional hypothesis testing typically assesses whether the $\Delta\Psi$ is nonzero without considering its magnitude.

rMATS accounts for the estimation uncertainty of individual replicates by modelling $\Psi$ values using a Binomial distribution and captures variability among replicates through a hierarchical mixture model. This framework simultaneously estimates both the effect size ($\Delta\Psi$) and the inherent variability across biological replicates. The statistical significance of differential splicing events is determined using a Likelihood Ratio Test (LRT), which compares the fit of models with and without the splicing difference. Therefore, rMATS identifies splicing events where the observed $\Delta\Psi$ is unlikely to have occurred by chance, thereby distinguishing biologically meaningful changes from random variations. rMATS also includes the possibility to perform an FDR correction.

$\Psi$ values in SUPPA2 are estimated based on transcript abundances, typically derived using quantification tools such as Kallisto. Once the transcript abundances are obtained, SUPPA2 calculates the observed $\Delta\Psi$ and the average abundance of each transcript under investigation. Using these metrics, SUPPA2 constructs an empirical cumulative distribution function (CDF) of transcripts with similar abundances, which serves as the foundation for determining p-values. These p-values represent the probability that the observed $\Delta\Psi$ could have occurred by chance under the null hypothesis of no differential splicing.

To account for multiple hypothesis testing, SUPPA2 adjusts the calculated p-values to control the False Discovery Rate (FDR), ensuring that the proportion of false positives remains low. One of SUPPA2's significant advantages is its computational efficiency. The tool is highly effective and operates at least an order of magnitude faster than other AS detection tools like MAJIQ, rMATS or EventPointer (both EPBAM and EPST), making it particularly suitable for large-scale RNA-Seq datasets. Unlike the other tools, SUPPA2 does not allow to set a threshold on ΔΨ within its core statistical framework. However, users have the flexibility to implement such thresholds post-analysis through filtering steps.

EventPointer (EPBAM and EPST), as both rMATS and SUPPA2, provides a p-value to state the statistical significance of AS events. The primary distinction between EventPointer and these tools lies in its statistical model. EventPointer employs bootstrap-based statistics to evaluate the significance of changes in $\Psi$ values ($\Delta\Psi$). Depending on the specific mode of operation, bootstrapped samples in EventPointer are generated either through transcript quantification tools like Kallisto (when using EPST) or internally produced using the reads mapped to an AS event (when using EPBAM). The characteristics of EventPointer and its two workflows, EPST and EPBAM, are detailed in Supplementary Text 6: Comparative Analysis of EPBAM and EPST Workflows.

EventPointer describes the experimental design using design and contrast matrices following a framework similar to limma (10). This feature enhances its versatility, allowing it to handle more complex experimental setups beyond simple case-control studies. It also allows for control for the presence of confounding factors. EP provides FDR correction using qvalue R package (11).

Shiba assesses differential splicing directly from pooled junction read counts, without modelling a distribution for Ψ or ΔΨ. For a given event, replicate junction reads are first pooled within each group; the resulting inclusion and exclusion counts are then arranged into one or more 2×2 contingency tables (two tables, for the upstream and downstream junctions, for skipped-exon and retained-intron events; a single table for events with one alternative junction, such as alternative 5'/3' splice sites and alternative first/last exons; four tables for mutually exclusive exons; n+1 tables for an event with n skipped exons). For each table, Shiba computes an odds ratio (OR) between the two groups and a Fisher's exact test p-value; p-values are corrected for multiple testing using the Benjamini–Hochberg procedure to obtain q-values. By default, an event is called a differentially spliced event (DSE) only if every OR exceeds a user-defined threshold and the largest of the associated q-values is below 0.05, so that all junctions contributing to the event must independently support the change.

Because Fisher's exact test is computed on pooled read counts rather than on a per-replicate Ψ estimate, this framework is, by construction, agnostic to the number of replicates and remains applicable to single-replicate designs (n = 1), unlike rMATS' hierarchical binomial model or the bootstrap-based procedures of EventPointer. This comes at the cost of ignoring biological variability between replicates. Shiba+ addresses this by additionally requiring a significant Welch's t-test (p < 0.05) on the per-sample Ψ values on top of Shiba's original criteria.

LeafCutter tests differential intron usage at the level of the whole cluster. For a cluster of J alternatively excised introns, the vector of per-sample read counts across the J introns is modelled jointly with a Dirichlet-multinomial generalised linear model, in which the group covariate is allowed to shift the multinomial proportions of the cluster; the implicit normalisation of the multinomial likelihood means that no separate library-size correction is required. Statistical significance is assessed with a likelihood ratio test comparing this model against a null model without the group covariate, using K − 1 degrees of freedom, where K is the number of introns in the cluster; this produces a single p-value per cluster, in contrast to the per-path or per-junction p-values reported by the other tools. Effect sizes (ΔΨ) are nonetheless reported for each intron individually. By default, only introns detected in at least five samples are tested, and a cluster is tested only if each group contains at least four samples with 20 or more spliced reads supporting introns in the cluster; both thresholds are user-customisable.

MAJIQ includes two methods to state the change in AS: $\Delta\Psi$ and HET. Both pipelines share the same underlying Bayesian model to infer the posterior distribution of $\Psi$. MAJIQ HET uses robust rank-based statistics (TNOM, InfoScore, or Mann–Whitney tests), combined with estimated $\Delta\Psi$ between the median of each group to filter and rank differential splicing events. We will focus on the $\Delta\Psi$ pipeline in this manuscript since it is more appropriate for case-control studies. Formally, $\Psi$ for a junction in an LSV is defined as the fraction of expressed isoforms using the junctions of all expressed isoforms containing the LSV. Each read can be modelled as a binomial distribution whose probability (unknown) is $\Psi$. Using a Bayesian approach, they include prior knowledge of $\Psi$, especially useful, for lowly expressed isoforms. The inference of the *a posteriori* $\Psi$ is performed using bootstrap. Once the distribution of Ψ is computed for each of the groups, MAJIQ $\Delta\Psi$ computes point estimates of ΔΨ using the posterior mean of the distribution ([$E[\Delta\Psi]$) and identifies confidence of measured changes in inclusion as posterior probabilities $P(\mid\Delta\Psi\mid> c)$.

MAJIQ and EventPointer are based on bootstrap estimation of the distribution of $\Delta\Psi$. However, its use of this distribution is quite different. MAJIQ requires two thresholds to estimate two probabilities: one to compute the probability of $\left| \Delta\Psi\right|$ is not changing (defaults to 0.05), and the other to compute the probability of $\left| \Delta\Psi\right|$ is changing (defaults to 0.2). EP exploits the close relationship between confidence intervals and p-values: the p-value is one minus the alpha level so that, the confidence interval for $\Delta\Psi$ critically includes the selected threshold (detailed in Supplementary Text 4: "P-value Computation for EventPointer").

In this article, to determine the significance of $\Delta\Psi$ for a MAJIQ event, a p-value estimation has been performed for each event based on the probabilities of change associated with each junction that constitutes an event. These estimated p-values for each junction were subsequently summarized using Fisher's method for combining probabilities (detailed in Supplementary Text 4: "P-value calculation for an AS event in MAJIQ+VOILA").

Figure S10 displays the estimated distribution of $\Delta\Psi$ in four different scenarios using both EP and MAJIQ. In the first event (panel A), most of the estimated distribution for $\Delta\Psi$ is positive and larger than the threshold. Therefore, the probability of change is large (around 0.8), and the probability of no-change is small (around 0.05). Both probabilities do not sum up one since the thresholds are different in each case. EventPointer provides a p-value of 0.72. In this case, the distribution of $\Delta\Psi$ has a large variance, and therefore, the p-value is non-significant.

In the second case (panel B), the distribution of $\Delta\Psi$ is negative (but small if compared with the threshold for changing) with a very small variance. As a consequence, both the probability of change and no-change are small (around 0). The p-value returned by EP will be close to zero if the threshold is very small (Figure S11), or close to one, if the threshold is larger as it happens in this case.

The third case (panel C) can be considered a prototypical case of a differential splicing event. In this case, the probability of change is close to one (0.947 in this case), the probability of no-change is small (0.001 in this case) and the p-value returned by EP is close to zero (0.037 in this case). In these cases, the p-value returned by EP is approximately equal to two times one minus the probability of change returned by MAJIQ if the threshold for EP and the probability of change is identical.

Finally, in the fourth scenario (panel D), the distribution of $\Delta\Psi$ is close to zero. In this case, the probability of change is close to zero, and the probability of no-change is large (0.475 in this case). The EP p-value will be close to one as well.

**Figure S10:** The figure illustrates the contrasting approaches of MAJIQ and EventPointer in determining splicing variation ($\Delta\Psi$) and statistical significance. The black line is the estimated distribution of $\Delta\Psi$. The density function spans from -1 to +1 and the area under this density function is one in all the cases. The probability of “changing” in MAJIQ is shown with orange lines (i.e. the probability of $\left| \Delta\Psi\right|$ being larger than a threshold. EventPointer’s p-value is shown as cyan lines. The red line is an auxiliary function used to compute the p-value.

**P-value calculation for an AS event in MAJIQ+VOILA**

We summarized the probability of being differentially spliced using the Fisher method (12).

Let $p_{i}$ denote the change probability of a junction associated with a splicing event.
The number of junctions for a given event is represented by $n$. To summarize the probabilities using Fisher's method for combining p-values. Assuming the p-values ($1-p_{i})$ , the Pearson method exploits that, if the p-values follow a uniform (0,1) distribution, the following statistics,

$X^{2}=-2\sum_{i=1}^{n} ln \left( 1-p_{i}+\epsilon\right)$,	(19)

follows a $X^{2}$ distribution with 2n degrees of freedom. A small constant $\epsilon$ ($\epsilon={10}^{-10}$) was added to avoid to computing the logarithm of zero. The summarized p-value is then calculated as:

$P=1-F_{\chi^{2}}\left( X^{2};2n \right),$	(20)

where $F_{\chi^{2}}\left( X^{2};2n \right)$ is the cumulative distribution function (CDF) of a chi-squared distribution with 2n degrees of freedom, evaluated at $X^{2}$.

This approach was used to combine the probabilities of individual junctions into a single p-value for the splicing event.

**P-Value Computation for EventPointer**

Since we estimate the distribution of the desired contrast rather than the null distribution, the p-value is determined using the relationship between confidence intervals and hypothesis testing (13). Once the contrast distribution is obtained, the p-value is computed by analyzing the tail areas of the density function. Specifically, the p-value is given by:

$p-value=2 (1-max(\int_{-\infty}^{0} f_{(x)}dx,\int_{0}^{\infty} f_{(x)}dx))$	(21)

where $f_{(x)}$ is the estimated distribution based on bootstrap simulation.

The null hypothesis (H0) can be defined by ∣ΔΨ∣<θ, where θ is a predefined threshold. In this case, the H0 is composite. We can consider the p-value using the least favorable case, focusing on the tail regions beyond θ. The p-value would be in this case:

$p-value=2 (1-max(\int_{-\infty}^{-\theta} f_{(x)}dx,\int_{\theta}^{\infty} f_{(x)}dx))$	(22)

If θ is large and the contrast distribution is centered around zero, the integral values may be negligible, leading to a p-value that could exceed 1 (with a maximum of 2). In such cases, the p-value is constrained to 1. Alternatively, we incorporate a correction factor that accounts for the area of the null hypothesis region. The adjusted formula is:

$p-value=\left( 2-\gamma\right)\left( 1-max\left( \int_{-\infty}^{-\theta} f_{(x)}dx,\int_{\theta}^{\infty} f_{(x)}dx \right) \right)$	(23)

where:

$\gamma=\int_{-\theta}^{\theta} f_{(x)} dx$,	(24)

This correction ensures that the computed p-value remains within the valid range of [0,1]. The $\gamma$ value is precisely the probability of differential splicing of MAJIQ if the threshold in MAJIQ and EP is selected to be identical. One advantage of this correction factor is that the distribution of the p-values does not have a spike at one, helping to rank the events even if the selected threshold is large. Figure S11 shows how the p-value is computed for the events described in the main text. It also illustrates the changes in the p-values when the threshold to declare an event to be significant is changed.

For these computations, EventPointer directly utilizes the estimated distribution of ΔΨ, distinguishing it from traditional methods that infer p-values by estimating the null distribution. Conceptually, this approach aligns with determining confidence intervals for ΔΨ at varying significance levels and selecting the smallest α that allows for rejecting the null hypothesis.

**Figure S11:** To illustrate the effect of the threshold, it is shown the p-value for EventPointer in the same cases of the main manuscript using different thresholds. The first column shows the area that corresponds to the p-value for a threshold equal to 0. The middle and the third column show the p-values when this threshold is set to 0.1 and 0.2 respectively. In all the cases increasing the threshold also increases the p-value. For example, in B case, the $\Delta\Psi$ is small but different from 0, increasing the threshold changes the p-value from 0.006 to 0.998.

**Supplementary Text 5: Simulated events**

**Classification of True Positives, True Negatives, False Negatives, and False Positives for simulated dataset**

Events were classified as follows:

- True Positives (TP): Positive events (ΔΨ > 0.2) detected by the tool and identified as significant (p-value < 0.05).
- True Negatives (TN): Negative events (ΔΨ ≤ 0.2) not detected or detected but classified as non-significant (p-value ≥ 0.05).
- False Negatives (FN): Positive events (ΔΨ > 0.2) either undetected or detected but classified as non-significant (p-value ≥ 0.05).
- False Positives (FP): Negative events (ΔΨ ≤ 0.2) detected and incorrectly classified as significant (p-value < 0.05).

These definitions were applied uniformly across all simulated splicing event types (Skipping Exons [SE], Alternative 3' Splice Sites [A3], and Alternative 5' Splice Sites [A5]) and sequencing depths (60M, 25M, 10M, 5M, 3M, and 1M reads).

For MAJIQ+VOILA, which does not report a p-value but a posterior probability of differential splicing, that value is the summarized quantity defined in Supplementary Text 4.

**Supplementary Text 6: Alignment Details**

The alignment of reads for novo splicing tools was performed using the STAR aligner, following the same command recommended by rMATS version 4.3 for its internal sequence alignment. Key parameters used in the alignment include:

- --chimSegmentMin 2: Sets the minimum number of bases required for chimeric junctions, enabling the detection of split reads mapping to different regions.
- --outFilterMismatchNmax 3: Allows a maximum of 3 mismatches per read, ensuring high-confidence alignments.
- --twopassMode Basic: Enables a two-pass alignment mode, improving splice junction detection by re-aligning reads after discovering junctions in the first pass.
- --runThreadN 10: Utilizes 10 threads to parallelize the alignment process, reducing computational time.
- --outSAMstrandField intronMotif: Includes strand information in the output BAM file for compatibility with downstream splicing analysis tools.
- --alignSJDBoverhangMin 1: Sets the minimum overhang for spliced alignments across splice junctions, allowing accurate detection of splice sites.
- --alignIntronMax 299999: Defines the maximum intron length for alignment, accommodating long introns typically found in human genes.

The choice of these parameters is critical for accurate detection of splicing events. Variations in their values can significantly influence the identification of splice junctions and the overall performance of downstream splicing analyses.

Further, the Supplementary Table S3 reports, for each tool, the annotation used, the parameters that depart from the tool's defaults, and the criterion by which an event was declared differentially spliced. Parameters not listed were left at their default values. Thresholds were deliberately not harmonised across tools; the rationale and the sensitivity analysis are given in Supplementary Text 10. Where a threshold is marked “default”, the tool was invoked without that flag and the value shown is the documented default for the version listed in Methods.

**Table S3:** Annotation, non-default parameters and calling criteria for each tool.

| **Tool** | **Annotation (real dataset)** | **Detection / quantification parameters** | **Statistical test** | **Calling criterion used here** |
| --- | --- | --- | --- | --- |
| rMATS | Ensembl 113 (Homo_sapiens.GRCh38.113.chr.gtf) | --readLength 100 (101 for SIRV); --allow-clipping; --nthread 8; --cstat, --novelSS, --mil/--mel and -t left at defaults; JCEC counts used | Likelihood-ratio test on inclusion levels | Nominal PValue < 0.05 |
| SUPPA2 | Ensembl 113 (Homo_sapiens.GRCh38.113.chr.gtf); kallisto v0.44.0, 100 bootstraps, index from Homo_sapiens.GRCh38.cdna.all (k = 31) | generateEvents -f ioe -e SE SS MX RI FL; strict boundaries (default), exon length 100 nt (default), threshold 10 nt (default); diffSplice --method empirical -th 0 -nan 0 -pa -gc | Empirical p-value from a within-condition null, corrected by gene (--gene-correction) | Nominal p-value < 0.05 (already gene-corrected) |
| MAJIQ + VOILA | Ensembl 113 GFF3 (Homo_sapiens.GRCh38.113.chr.gff3) | majiq build with minreads, minpos and min_experiments at defaults; majiq deltapsi at defaults; voila modulize at defaults | Posterior probability that \|ΔΨ\| exceeds a fixed effect size | VOILA defaults: changing threshold \|ΔΨ\| = 0.20, probability_changing = 0.95. No significance filter applied by us |
| EPBAM | Ensembl 113 (Homo_sapiens.GRCh38.113.chr.gtf) | min_junction_count = 2; min_anchor = 6; max_complexity = 30; min_n_sample = NULL; nboot = 20; μ estimated from the data | Two-level bootstrap p-value | Nominal p-value < 0.05 (local FDR and q-values are computed but were not used) |
| EPST | Ensembl 113 (Homo_sapiens.GRCh38.113.chr.gtf); kallisto v0.44.0, 100 bootstraps, index from Homo_sapiens.GRCh38.cdna.all (k = 31) | Bootstrap = TRUE; Filter = FALSE; Qn = 0.25 | Two-level bootstrap p-value | Nominal p-value < 0.05 |
| LeafCutter | Ensembl 113 (Homo_sapiens.GRCh38.113.gtf) | regtools junctions extract -a 8 -m 50 -M 500000 -s XS; leafcutter_cluster_regtools.py -m 50 -l 500000 -k 1; leafcutter_ds.R with min_samples_per_intron, min_samples_per_group and min_coverage at defaults | Dirichlet-multinomial likelihood-ratio test, per intron cluster | Nominal cluster-level p-value < 0.05 (the Benjamini-Hochberg value reported by the tool was not used) |
| Shiba | Ensembl 113 (Homo_sapiens.GRCh38.113.gtf); MameShiba mode (--mame), i.e. without de novo transcript assembly | minimum_anchor_length 6; minimum_intron_length 70; maximum_intron_length 500000; minimum_reads 10; strand XS; unannotated True; individual_psi True; ttest True | Fisher exact test on junction counts, plus Welch t-test on Ψ | Tool defaults declared in the configuration file: FDR < 0.05 and \|ΔΨ\| > 0.1 |

Differences between datasets: for the SIRV benchmark, rMATS used --readLength 101 and EventPointer used min_n_sample = 3, and all tools used the SIRV-extended Ensembl 90 reference; all other parameters were as above. The simulated dataset was analysed against the RefSeq hg19 annotation on the GRCh37 assembly; reads were taken from the published SUPPA2 benchmark and were not simulated by us.

**Supplementary Text 7: Calibration of EPBAM's bootstrap inference**

EPBAM's inference proceeds in two stages: a read-level bootstrap within each sample, which yields a distribution of Ψ per sample, and a second-level resampling across samples, which yields the distribution of ΔΨ from which p-values are obtained. To characterise the calibration of the resulting p-values and q-values we used three settings in which the true ΔΨ is known to be zero.

(i) Null contrasts in SIRV spike-in data. The SIRV dataset comprises 20 samples per Mix, all carrying an identical spike-in. Any split of the samples of a single Mix into two arbitrary groups therefore has a true ΔΨ of exactly zero for every SIRV event, while retaining the sample-to-sample heterogeneity of real libraries. We generated 100 random balanced splits per Mix at group sizes n = 2, 3, 5 and 10, giving 85,200 p-values under a complete null.

(ii) Within-condition splits in the CX-4945 dataset. Since SIRV spike-ins are added at fixed concentration, setting (i) characterises a regime of low between-replicate dispersion. To probe biological replicate variability we split the five replicates of a single cell line and a single condition into arbitrary pseudo-groups, exhaustively over all six (cell line × condition) strata and both 2-versus-2 and 2-versus-3 designs, giving 300,000 p-values under a complete null with full biological variability.

(iii) Label permutation. We recomputed the CX-4945 contrast under 200 permutations of the condition labels, restricted within cell line. In this dataset the permutation null is conservative by construction, because the drug effect is large enough that permuted pseudo-groups mix treated and vehicle samples; setting (ii) is therefore the primary evidence and (iii) a supporting, one-sided check. All analyses use the default θ = 0.

**Table S4: Calibration of EPBAM's p-values and q-values under a complete null. λ is the χ²₁ inflation factor (1 = calibrated, below 1 = conservative); π̂₀ is Storey's estimate of the proportion of true nulls; m is the number of p-values.**

| **Setting** | **n per group** | **m** | **type-I α=0.01** | **α=0.05** | **α=0.10** | **λ** | **π̂₀** | **q<0.05** |
| --- | --- | --- | --- | --- | --- | --- | --- | --- |
| SIRV null splits | 2 | 21,300 | 0.0084 | 0.0267 | 0.0495 | 0.38 | 1.00 | 20 |
| SIRV null splits | 3 | 21,300 | 0.0046 | 0.0175 | 0.0352 | 0.35 | 1.00 | 4 |
| SIRV null splits | 5 | 21,300 | 0.0029 | 0.0114 | 0.0276 | 0.33 | 1.00 | 0 |
| SIRV null splits | 10 | 21,300 | 0.0011 | 0.0074 | 0.0202 | 0.31 | 1.00 | 0 |
| CX-4945 within-condition (drug) | 2 | 90,000 | 0.0102 | 0.0337 | 0.0606 | 0.52 | 1.00 | 78 |
| CX-4945 within-condition (vehicle) | 2 | 90,000 | 0.0099 | 0.0316 | 0.0575 | 0.30 | 1.00 | 59 |
| CX-4945 within-condition (drug) | 2 vs 3 | 60,000 | 0.0087 | 0.0296 | 0.0567 | 0.50 | 1.00 | 41 |
| CX-4945 within-condition (vehicle) | 2 vs 3 | 60,000 | 0.0085 | 0.0288 | 0.0579 | 0.31 | 1.00 | 28 |
| CX-4945 label permutation | 15 | 400,000 | 0.0051 | 0.0206 | 0.0446 | 0.52 | 1.00 | 172 |

The type-I error is at or below the nominal level at every level and group size tested, in every setting. π̂₀ equals 1.00 throughout, and Storey's q-values and Benjamini–Hochberg gave identical numbers of calls. Under a complete null the realised proportion of discoveries at q < 0.05 was 0.03% for the SIRV splits and 0.07% for the within-condition splits. Results were consistent across the three SIRV Mixes and across the three cell lines (type-I at α = 0.05 of 0.0329, 0.0296 and 0.0313). The p-value distribution is conservative rather than exactly uniform (Kolmogorov–Smirnov D ≈ 0.21 for the SIRV splits): the departure is towards larger p-values.

A comparison at matched group size is informative. With n = 2, the type-I error at α = 0.05 was 0.0267 in the SIRV setting, where between-replicate dispersion is negligible, and 0.0316–0.0337 in the within-condition splits of the CX-4945 data, which carry full biological variability. Control is therefore retained under genuine biological replicate variability, and calibration moves closer to nominal as that variability increases.

**Figure S12: Histograms of nominal p-values and quantile–quantile plots against the uniform distribution for the SIRV null contrasts, by group size.**

**Figure S13: The same read-outs for the within-condition splits of the CX-4945 dataset, by cell line.**

**Origin of the conservatism**. Write the observed Ψ of sample i as Ψᵢ = ψ_group + bᵢ + eᵢ, where bᵢ is the between-replicate departure (variance σ²_b) and eᵢ the read-sampling error (variance σ²_e). The sampling variance of the contrast for n samples per group is 2(σ²_b + σ²_e)/n. Each second-level value is drawn from the pooled set of bootstrap replicates of the samples of a class. That pool carries two contributions: the spread of the per-sample point estimates, which is σ²_b + σ²_e, and the within-sample bootstrap spread, which is σ²_e again. The bootstrap variance is therefore 2(σ²_b + 2σ²_e)/n, one unit of read-sampling variance in excess. This is the known behaviour of a naive nested bootstrap, in which resampling within a unit after having resampled the units themselves counts the within-unit variance twice.

Simulation with the between-replicate component set to zero confirms the prediction: the ratio of the bootstrap standard deviation of ΔΨ to its true sampling standard deviation was 1.14, 1.23, 1.28 and 1.35 at n = 2, 3, 5 and 10, approaching the predicted √2. The model also accounts for two features of the empirical results: conservatism increases with n, and it is milder in the drug-treated samples, where between-replicate variability is larger and the excess technical term therefore weighs less. The practical consequence is a loss of power rather than an inflation of false positives.

**Limitations and scope.** At three replicates per group the resampling is necessarily coarse and we recommend n ≥ 5. All results use θ = 0, a point null; this is also the setting in which the results above are interpretable, since the null contrasts have ΔΨ = 0 exactly, which lies on the boundary of the null hypothesis when θ = 0, so that uniformity of the p-value is the correct benchmark. Users who wish to impose a minimum effect size should set θ > 0 rather than filter on |ΔΨ| after testing against θ = 0, since the latter does not preserve FDR control for the filtered set; the resulting test of the interval null |ΔΨ| ≤ θ is valid but necessarily conservative for events lying well inside the interval.

We also examined how the residual conservatism could be removed. Subtracting the duplicated variance component directly is unstable, as it requires the difference of two noisy variance estimates. A studentized (bootstrap-t) formulation is preferable since it uses a pivotal statistic: a constant inflation of the variance appears in both the statistic and its standard error and therefore cancels without needing to be estimated. Combining this with empirical-Bayes shrinkage of the variance across events is a natural further step. We regard these as improvements to the method rather than corrections; the inference reported here is valid, if conservative, as it stands.

**A note on multifactor designs**. The second-level resampling groups samples by their weight in the contrast rather than by their row in the design matrix. For a two-group design the two are identical, and all analyses in this study — SIRV, simulated data, and the per-cell-line CX-4945 contrasts — use two-group designs. For a design containing nuisance factors they differ: in a balanced design with three cell lines and two conditions the contrast weight takes only two values, so all treated samples are pooled irrespective of cell line and the between-cell-line variance enters the null distribution. The point estimate of ΔΨ remains correctly adjusted; the reference distribution does not. The effect is conservative: in these data the type-I error at α = 0.05 fell from 0.032 to 0.011 when the three cell lines were combined at matched per-line replication, so that tripling the sample size made the test less sensitive. Pooling by design row has been implemented in EventPointer, with a minimum-replication safeguard, since with fewer than about five replicates per design cell the smaller pools make the procedure mildly anti-conservative.

**Supplementary Text 8: Computational requirements**

Table S5 reports the computational cost of each tool for the global CX-4945 versus DMSO comparison, with all three TNBC cell lines pooled (n = 15 per group). This pooled configuration is the heavier one and is reported here as the fair basis for a cost comparison, although the differential results in the main text are obtained per cell line. Times start from aligned BAM files; the STAR alignment step is shared across all methods (but SUPPA that uses kallisto) and is not included. CPU efficiency is CPU-time utilised divided by CPU-time allocated, and peak RAM is the maximum across pipeline steps. Sample assignments were confirmed from each tool's own sample sheet. The comparison covers six of the seven tools. EPST is not included: it shares the kallisto quantification step with SUPPA2, and its remaining steps were not timed separately.

**Table S5: Runtime, CPU efficiency and peak memory for each tool.**

| **Tool** | **Steps** | **Wall-clock** | **CPUs** | **CPU efficiency** | **Peak RAM** |
| --- | --- | --- | --- | --- | --- |
| rMATS | single job | 52 m 26 s | 8 | 82.1% | 5.73 GB |
| MAJIQ | build (30) + quantify (30) + voila categorize (8) | ~55 m 48 s | 8–30 | 19.1% | 1.71 GB |
| SUPPA2 | events + PSI + join + join TPM + diffSplice | ~3 h 39 m 27 s | 1 | 28.6% | 1.62 GB |
| EventPointer (EPBAM) | single job | 27 h 46 m 08 s | 10 | 78.2% | 55.33 GB |
| LeafCutter | bam2junc + gtf2exons + cluster + ds (4) | ~1 h 51 m 39 s | 1–4 | 84.4% | 2.47 GB |
| Shiba | single job | 7 h 02 m 48 s | 4 | 34.8% | 12.69 GB |

EPBAM is substantially more demanding than the other methods, requiring 27.8 h and 55 GB of peak memory against 52 min and 5.7 GB for rMATS. Two design choices account for this. First, EPBAM constructs the full splice graph from the aligned reads rather than restricting itself to an annotated set of events, so its cost scales with the complexity of the observed transcriptome rather than with the size of the annotation. Second, the read-level bootstrap re-estimates Ψ, and with it the coverage-correction coefficients, for every replicate of every event, which is the price of the uncertainty estimates that the statistical framework requires. The memory footprint is dominated by holding the splice graph and the bootstrap array for all events simultaneously. These costs are incurred once per dataset —the detection and bootstrap stage is computed once and reused for every contrast— but they place EPBAM at the expensive end of the tools evaluated here, and users working on large cohorts should plan accordingly. Processing the splice graph in chromosome-wise blocks would reduce the memory footprint substantially and is a straightforward optimisation that we have not yet implemented.

**Supplementary Text 9: Sensitivity to the regularisation parameter λ**

The coefficients u and v are estimated per event from all samples jointly by penalised non-negative least squares, with a penalty that pulls the solution towards the classical effective-length ratios. The penalty weight is scaled so that the penalty term is worth a fixed fraction λ of the residual deviance of the unregularised fit, which makes λ dimensionless and comparable across events of very different expression. We assessed the sensitivity of u, v, Ψ and ΔΨ to λ by simulation. Counts for the two paths and the reference path were generated for two groups of n samples at a given locus depth, with true coefficients (u, v) = (1.4, 0.7) — that is, with the classical effective-length assumption wrong by 40% and 30% respectively — and with the true ΔΨ varied from 0 to 0.6. Two hundred replicates per cell.

**Table S6: Sensitivity of the coverage-correction coefficients and of Ψ and ΔΨ to λ. Means over 200 simulated events with n = 5 per group and 1,000 reads at the locus; bias and standard deviation of û are relative to the true u.**

| **λ** | **true ΔΨ** | **bias(û)** | **sd(û)** | **RMSE(Ψ)** | **bias(ΔΨ)** |
| --- | --- | --- | --- | --- | --- |
| 0 | 0.0 | −0.325 | 0.454 | 0.233 | +0.0002 |
| 0 | 0.2 | +0.0000 | 0.075 | 0.034 | −0.0012 |
| 0 | 0.6 | −0.0015 | 0.031 | 0.015 | −0.0000 |
| 0.1 | 0.0 | −0.277 | 0.343 | 0.189 | −0.0005 |
| 0.1 | 0.2 | −0.011 | 0.088 | 0.039 | −0.0004 |
| 0.1 | 0.6 | −0.005 | 0.030 | 0.016 | −0.0010 |
| 1 | 0.0 | −0.312 | 0.123 | 0.162 | +0.0011 |
| 1 | 0.2 | −0.085 | 0.085 | 0.052 | −0.0014 |
| 1 | 0.6 | −0.014 | 0.029 | 0.016 | +0.0003 |

The coefficients are identifiable only when Ψ varies across samples. If every sample has the same Ψ, the two path signals are proportional to one another, the design matrix is effectively rank-deficient, and only the combination u·S₁ + v·S₂ is determined; u and v individually are not. This is visible in the first row of each block: at ΔΨ = 0 the relative standard deviation of û is 0.45 with no regularisation, and it does not improve with sequencing depth (0.48, 0.44 and 0.45 at 100, 1,000 and 10,000 reads). As soon as Ψ varies the coefficients become well determined: the relative bias of û falls from −0.33 at ΔΨ = 0 to −0.04 at ΔΨ = 0.1 and below 0.005 for ΔΨ ≥ 0.2.

Strong differential usage therefore does not bias the correction factors; it is what makes them estimable. The regime that requires care is the opposite one, events whose Ψ is nearly constant across samples, and it is precisely there that the penalty is doing useful work: increasing λ from 0 to 1 reduces the RMSE of Ψ from 0.233 to 0.162 at ΔΨ = 0, while changing it by less than 0.001 at ΔΨ = 0.6. We found a similar behaviour when we developed EventPointer for junction arrays.

Estimates of ΔΨ are insensitive to λ throughout. The bias of ΔΨ remains below 0.005 in every cell of the grid, across all values of λ, of the true ΔΨ, of sequencing depth (100–10,000 reads) and of replicate number (n = 3, 5, 10). Errors in û and v̂ largely cancel in the difference between groups, so the quantity on which the differential analysis rests is robust to the choice of λ even where the coefficients themselves are poorly determined. Leave-one-sample-out cross-validation selects λ ≈ 0 when Ψ varies across samples and λ between 0.3 and 3 when it does not, bracketing the default of 0.1.

These simulations vary the coefficients away from the classical effective-length ratios but assume, as the model does, that a single pair (u, v) applies to all samples of an event. They therefore characterise estimation error under a correctly specified model, and do not address misspecification arising from coverage bias that differs between libraries or systematically between conditions. The read-level bootstrap propagates the estimation uncertainty of u and v but not the consequences of a misspecified coverage model.

**Figure S14.** Per-event Ψ profiles for the 23 RT-PCR-validated events of the CX-4945 dataset. Each panel shows one gene; the x axis lists the 30 RNA-seq samples (15 DMSO, then 15 CX-4945, separated by the dashed vertical line) and the y axis the Ψ value. The red dashed series is the RT-PCR measurement (available for two replicates per cell line and condition); the remaining series are the estimates of EPBAM, EPST, rMATS, SUPPA2, MAJIQ+VOILA, LeafCutter and Shiba. Point shape distinguishes annotation-based from non-annotation tools. Series are shown only for the tools that detected the event; where a tool measured the complementary path, its Ψ was inverted so that all series are on a comparable scale.

**Supplementary Text 10. Sensitivity of the benchmark to the decision thresholds**

We interrogated whether our conclusions depend on the particular thresholds used on the SIRV spike-ins. The analysis re-filters the per-event results already reported; no tool was re-run, so it is internally consistent with the rest of the manuscript.

We swept the minimum effect size, |ΔΨ| from 0 to 0.30, crossed with the significance cut-off (p < 0.01, 0.05 and 0.10), under each of the three annotation conditions. An event was counted as a genuine change when the SIRV molar design implies |ΔΨ| ≥ 0.10, and as non-changing when it implies |ΔΨ| < 0.05. Events falling between those two values were excluded, so that no method is penalised for ambiguous cases. Table S7 reports the observed FDR at p < 0.05 and Figure S15 shows it graphically; Figure S16 shows the corresponding number of true positives. The complete grid is provided as a machine-readable file.

Two results deserve to be stated plainly. First, the observed FDR decreases monotonically with the effect-size threshold for every method except MAJIQ+VOILA, whose FDR remains essentially flat (0.05–0.09) in the complete and overloaded conditions. Its false positives are therefore not small-effect calls that a stricter threshold would remove: they are calls with a large estimated ΔΨ that do not correspond to a real change, which is consistent with the false positives reported for MAJIQ elsewhere in the SIRV benchmark. Second, the ranking of the methods is not invariant across the whole range: with no effect-size filter rMATS attains the lowest FDR, whereas from |ΔΨ| ≥ 0.15 onwards EPBAM does. We report this rather than claim a stability the data do not support. What is stable is the substantive conclusion: at every threshold combination and in every annotation condition EPBAM remains among the methods with the lowest FDR, and its advantage under an incomplete annotation is preserved throughout. EPST, by contrast, reaches an FDR of 0.28 in that condition when no effect-size filter is applied.

A nominal p-value does not carry the same meaning across these tools. SUPPA2's p-value is empirical and bounded, so p < 0.05 is considerably more stringent on its scale than on that of a parametric test. We therefore repeated the comparison at an equal number of calls per tool, which removes the dependence on the scale of each statistic: for each method we ranked events by significance and examined the N most significant ones (Table S8, Figure S17). At matched numbers of calls the annotation-based methods are highly precise — EPST and SUPPA2 produce no false positives up to 100 calls in the complete condition — whereas MAJIQ+VOILA shows the highest FDR at every budget. This comparison is itself limited by the fact that the methods differ in how many true events they recover at all, so at large N some have exhausted their true positives while others have not.

Two limitations should be noted. MAJIQ+VOILA's statistic embeds its own effect-size threshold: it reports P(|ΔΨ| ≥ τ) with τ = 0.20 fixed at run time. We sweep the point estimate of ΔΨ that VOILA reports, but τ itself cannot be swept without re-running MAJIQ ΔΨ, so the MAJIQ curves should be read as a sweep of the reported effect size at a fixed internal τ. In addition, the number of matched events per tool and condition ranges from 19 to 75, so the curves are necessarily step-like and small differences between methods should not be over-interpreted. LeafCutter and Shiba are not included: their events are not mapped onto the SIRV ground truth, and constructing that mapping reliably was beyond the scope of this revision.

**Table S7. Observed FDR on the SIRV spike-ins as a function of the minimum |ΔΨ| threshold, at p < 0.05, for each tool and annotation condition.**

| **Tool** | **Annotation** | **\|ΔΨ\| ≥ 0** | **\|ΔΨ\| ≥ 0.05** | **\|ΔΨ\| ≥ 0.1** | **\|ΔΨ\| ≥ 0.15** | **\|ΔΨ\| ≥ 0.2** | **\|ΔΨ\| ≥ 0.3** |
| --- | --- | --- | --- | --- | --- | --- | --- |
| EPBAM | complete | 0.102 | 0.055 | 0.026 | 0.000 | 0.000 | 0.000 |
| EPBAM | incomplete | 0.126 | 0.049 | 0.027 | 0.017 | 0.000 | 0.000 |
| EPBAM | overloaded | 0.111 | 0.055 | 0.026 | 0.000 | 0.000 | 0.000 |
| EPST | complete | 0.154 | 0.033 | 0.027 | 0.022 | 0.000 | 0.000 |
| EPST | incomplete | 0.278 | 0.212 | 0.104 | 0.069 | 0.020 | 0.000 |
| EPST | overloaded | 0.253 | 0.051 | 0.029 | 0.023 | 0.000 | 0.000 |
| MAJIQ | complete | 0.088 | 0.088 | 0.056 | 0.058 | 0.062 | 0.054 |
| MAJIQ | incomplete | 0.087 | 0.087 | 0.024 | 0.000 | 0.000 | 0.000 |
| MAJIQ | overloaded | 0.088 | 0.088 | 0.056 | 0.058 | 0.062 | 0.054 |
| rMATS | complete | 0.073 | 0.022 | 0.000 | 0.000 | 0.000 | 0.000 |
| rMATS | incomplete | 0.152 | 0.032 | 0.000 | 0.000 | 0.000 | 0.000 |
| rMATS | overloaded | 0.137 | 0.036 | 0.022 | 0.030 | 0.043 | 0.000 |
| SUPPA2 | complete | 0.114 | 0.077 | 0.028 | 0.011 | 0.000 | 0.000 |
| SUPPA2 | incomplete | 0.175 | 0.130 | 0.043 | 0.000 | 0.000 | 0.000 |
| SUPPA2 | overloaded | 0.113 | 0.060 | 0.028 | 0.012 | 0.000 | 0.000 |

**Table S8. Observed FDR at an equal number of calls per tool (the N most significant events).**

| **Tool** | **Annotation** | **N = 10** | **N = 20** | **N = 30** | **N = 40** | **N = 50** | **N = 75** | **N = 100** |
| --- | --- | --- | --- | --- | --- | --- | --- | --- |
| EPBAM | complete | 0.000 | 0.000 | 0.000 | 0.000 | 0.040 | 0.053 | 0.120 |
| EPBAM | incomplete | 0.000 | 0.000 | 0.000 | 0.050 | 0.060 | 0.053 | 0.150 |
| EPBAM | overloaded | 0.000 | 0.000 | 0.000 | 0.000 | 0.040 | 0.053 | 0.120 |
| EPST | complete | 0.000 | 0.000 | 0.000 | 0.000 | 0.000 | 0.000 | 0.000 |
| EPST | incomplete | 0.000 | 0.000 | 0.000 | 0.000 | 0.000 | 0.173 | — |
| EPST | overloaded | 0.000 | 0.000 | 0.000 | 0.000 | 0.000 | 0.000 | 0.000 |
| MAJIQ | complete | 0.100 | 0.100 | 0.067 | 0.075 | 0.080 | 0.160 | — |
| MAJIQ | incomplete | 0.200 | 0.100 | 0.067 | 0.050 | 0.100 | — | — |
| MAJIQ | overloaded | 0.100 | 0.100 | 0.067 | 0.075 | 0.080 | 0.160 | — |
| rMATS | complete | 0.000 | 0.000 | 0.000 | 0.050 | 0.060 | — | — |
| rMATS | incomplete | 0.000 | 0.000 | 0.067 | 0.100 | 0.180 | — | — |
| rMATS | overloaded | 0.000 | 0.000 | 0.000 | 0.025 | 0.080 | 0.160 | — |
| SUPPA2 | complete | 0.000 | 0.000 | 0.000 | 0.000 | 0.000 | 0.000 | 0.010 |
| SUPPA2 | incomplete | 0.000 | 0.000 | 0.000 | 0.075 | 0.120 | — | — |
| SUPPA2 | overloaded | 0.000 | 0.000 | 0.000 | 0.000 | 0.000 | 0.000 | 0.010 |

Figure S15. Observed FDR on the SIRV spike-ins as a function of the minimum |ΔΨ| threshold (p < 0.05), for each annotation condition. All methods except MAJIQ+VOILA show a monotonic decrease.

Figure S16. Number of true positives recovered as a function of the minimum |ΔΨ| threshold (p < 0.05).

Figure S17. Observed FDR when each tool is allowed the same number of calls, removing the dependence on the scale of each statistic.

**Supplementary Text 11: Contribution of the coverage-corrected effective-length estimation**

The scaling coefficients u and v are estimated per event from the data, with the reference path acting as an additional constraint, by the penalised non-negative least-squares system of Equations (14)–(16), and Ψ follows from Equation (18). The regularisation parameter λ, set to 0.1 by default, controls how strongly that estimate is pulled towards the classical effective-length ratios: as noted after Equation (15), when λ is very large u and v converge to their priors l_Ref/l_A and l_Ref/l_B, and Equation (18) reduces to Equation (2) — the conventional effective-length correction used by alignment-based tools, which assumes uniform coverage along each path. The two limits of λ therefore correspond to estimating the coverage correction and to not estimating it at all.

We recomputed EPBAM pipeline for the SIRV spike-ins under the three annotation conditions (CA, IA and OA) using the conventional effective-length correction of Equation (2) and compared the result with the default EPBAM calculation (λ = 0.1), which is the one reported for EPBAM in Table 4 of the main text. Accuracy was measured on the events matched to the SIRV isoforms (48, 45 and 48 under CA, IA and OA). Table S9 reports the comparison. In addition to Table 4 of the main manuscript, Table S9 includes the mean signed error of Ψ and the MAE(Ψ). The former is computed as the average of Ψ− Ψ_SIRV over all matched events and samples measuring systematic bias, where a positive value indicates overestimation of Ψ, and the latter as the average of |Ψ− Ψ_SIRV| over the same values.

**Table S9: Ablation of the coverage-corrected effective-length estimation on the SIRV spike-ins. Each pair of columns compares the estimator implemented in EPBAM (coverage-corrected: u and v estimated by penalised non-negative least squares, λ = 0.1) with the conventional effective-length correction (u = lRef/lA and v = lRef/lB held fixed) under the complete (CA), incomplete (IA) and overloaded (OA) annotations. ρ is the Pearson correlation with the SIRV reference and RMSE the root mean square error; the mean signed error and the mean absolute error (MAE) of Ψ are computed over all matched events and samples. The coverage-corrected columns reproduce the values reported for EPBAM in Table 4 of the main text. The coverage-corrected method always outperforms the conventional.**

| **Metric** | **CA coverage-corrected** | **CA conventional** | **IA coverage-corrected** | **IA conventional** | **OA coverage-corrected** | **OA conventional** |
| --- | --- | --- | --- | --- | --- | --- |
| **Matched events** | 48 | 48 | 45 | 45 | 48 | 48 |
| **ρ(Ψ, ΨSIRV)** | **0.730** | 0.704 | **0.699** | 0.667 | **0.758** | 0.729 |
| **RMSE(Ψ)** | **0.242** | 0.252 | **0.258** | 0.270 | **0.229** | 0.239 |
| **Mean signed error of Ψ** | **0.027** | 0.087 | **0.025** | 0.090 | **0.018** | 0.077 |
| **MAE(Ψ)** | **0.152** | 0.190 | **0.176** | 0.216 | **0.144** | 0.182 |
| **ρ(ΔΨ, ΔΨSIRV)** | **0.898** | 0.868 | **0.912** | 0.827 | **0.947** | 0.891 |
| **RMSE(ΔΨ)** | **0.137** | 0.163 | **0.122** | 0.177 | **0.098** | 0.148 |

The effect of estimating u and v is essentially a reduction of bias: the mean signed error of Ψ falls from 0.087, 0.090 and 0.077 to 0.027, 0.025 and 0.018 under CA, IA and OA, with no appreciable change in the dispersion of the error. The gain is larger for ΔΨ (Δρ = 0.030, 0.085 and 0.056), which may seem to contradict the sensitivity analysis of Supplementary Text 9, where the value of λ was found not to affect ΔΨ. Notice that the two analyses answer different questions: Supplementary Text 9 varies λ within its working range, where u and v are still estimated from the data, whereas the present ablation removes the correction altogether. Therefore, whether the correction is applied does affect ΔΨ; which particular value of λ is used does not. The correction therefore improves the accuracy of both Ψ and ΔΨ.

**SUPPLEMENTARY REFERENCES**

1. Zhang,Y., Qian,J., Gu,C. and Yang,Y. (2021) Alternative splicing and cancer: a systematic review. *Signal Transduct. Target. Ther.*, **6**, 78.

2. Shen,S., Park,J.W., Lu,Z., Lin,L., Henry,M.D., Wu,Y.N., Zhou,Q. and Xing,Y. (2014) rMATS: Robust and flexible detection of differential alternative splicing from replicate RNA-Seq data. *Proceedings of the National Academy of Sciences*, **111**.

3. Trincado,J.L., Entizne,J.C., Hysenaj,G., Singh,B., Skalic,M., Elliott,D.J. and Eyras,E. (2018) SUPPA2: fast, accurate, and uncertainty-aware differential splicing analysis across multiple conditions. *Genome Biol.*, **19**, 40.

4. Romero,J.P., Muniategui,A., De Miguel,F.J., Aramburu,A., Montuenga,L., Pio,R. and Rubio,A. (2016) EventPointer: an effective identification of alternative splicing events using junction arrays. *BMC Genomics*, **17**, 467.

5. Ferrer-Bonsoms,J.A., Gimeno,M., Olaverri,D., Sacristan,P., Lobato,C., Castilla,C., Carazo,F. and Rubio,A. (2022) EventPointer 3.0: flexible and accurate splicing analysis that includes studying the differential usage of protein-domains. *NAR Genom. Bioinform.*, **4**, lqac067.

6. Green,C.J., Gazzara,M.R. and Barash,Y. (2018) MAJIQ-SPEL: web-tool to interrogate classical and complex splicing variations from RNA-Seq data. *Bioinformatics*, **34**, 300–302.

7. Vaquero-Garcia,J., Barrera,A., Gazzara,M.R., González-Vallinas,J., Lahens,N.F., Hogenesch,J.B., Lynch,K.W. and Barash,Y. (2016) A new view of transcriptome complexity and regulation through the lens of local splicing variations. *Elife*, **5**.

8. Lawrence,M., Huber,W., Pagès,H., Aboyoun,P., Carlson,M., Gentleman,R., Morgan,M.T. and Carey,V.J. (2013) Software for Computing and Annotating Genomic Ranges. *PLoS Comput. Biol.*, **9**, e1003118.

9. Lawson,C.L. and Hanson,R.J. (1995) Solving Least Squares Problems. *Solving Least Squares Problems*, 10.1137/1.9781611971217.

10. Smyth,G.K. (2005) limma: Linear Models for Microarray Data. *Bioinformatics and Computational Biology Solutions Using R and Bioconductor*, 10.1007/0-387-29362-0_23.

11. R Core Team (2025) stats-package: The R Stats Package.

12. Storey,J., Bass,A., Dabney,A. and Robinson,D. (2024) qvalue: Q-value estimation for false discovery rate control. R package version 2.38.0.

13. Strimmer,K. (2008) fdrtool: a versatile R package for estimating local and tail area-based false discovery rates. *Bioinformatics*, **24**, 1461–1462.

14. Fisher,R.A. (1992) Statistical Methods for Research Workers. 10.1007/978-1-4612-4380-9_6.

15. Panaretos,V.M. (2016) Statistics for mathematicians. *Compact Textbook in Mathematics. Birkhäuser/Springer*, **142**, 9–15.
